# Biocatalytic sulfation of aromatic and aliphatic alcohols catalyzed by arylsulfate sulfotransferases

**DOI:** 10.1007/s00253-024-13354-5

**Published:** 2024-11-19

**Authors:** Isabel Oroz-Guinea, Marko Rath, Isabelle Tischler, Klaus Ditrich, Doreen Schachtschabel, Michael Breuer, Wolfgang Kroutil

**Affiliations:** 1 Austrian Centre of Industrial Biotechnology C/o University of Graz, Heinrichstrasse 28, 8010 Graz, Austria; 2 Institute of Chemistry, University of Graz, NAWI Graz, Heinrichstrasse 28, 8010 Graz, Austria; 3 Field of Excellence BioHealth, University of Graz, Heinrichstrasse 28, 8010 Graz, Austria; 4 White Biotechnology Research, BASF SE, Carl-Bosch-Strasse 38, 67056 Ludwigshafen, Germany; 5 BioTechMed Graz, Heinrichstrasse 28, 8010 Graz, Austria

**Keywords:** Arylsulfate sulfotransferase, Enzymatic sulfation, Aliphatic alcohol sulfation, Amine sulfation, Disulfated compounds

## Abstract

**Abstract:**

Many relevant metabolites, as well as chemical commodities, contain at least one sulfate ester group. Consequently, biocatalytic strategies to attach sulfate to a molecule under mild conditions are of high interest. In order to expand the enzymatic toolbox available, five new arylsulfate sulfotransferases (ASSTs) were identified in this study. Overexpression in *Escherichia coli* and enzyme purification resulted in soluble proteins which catalyzed the sulfate transfer to an acceptor substrate using *p*-nitrophenyl sulfate (*p*NPS) as sulfate donor. Optimal reaction conditions were established with respect to temperature and pH, as well as their tolerance to organic co-solvents and melting temperature. Additionally, the kinetic parameters (*V*_max_, *K*_M_, and *k*_cat_) were determined. The substrate scope for the acceptor showed that a structurally diverse spectrum of alcohols is accepted. The substrates included phenolic alcohols with one, two, and three hydroxy groups, linear and cyclic aliphatic alcohols, and amines. The phenolic substrates were accepted reaching activities of up to 154 U/mg purified enzyme. Additionally, also the aliphatic alcohols (both linear and cyclic) were accepted at reduced activity, showing that these enzymes are not limited to phenolic alcohols. Moreover, catalytic activity was detected when using aniline as an acceptor substrate implying their ability to sulfate also amino groups. Finally, the consecutive sulfation of di- and trihydroxy compounds was observed, resulting in the detection of the corresponding disulfated molecules.

**Key points:**

• *Five novel arylsulfate sulfotransferases were identified and characterized.*

• *Accepted substrates included aromatic and aliphatic alcohols, as well as aniline.*

• *Disulfation of di- and trihydroxy aromatic compounds was studied and confirmed.*

**Supplementary Information:**

The online version contains supplementary material available at 10.1007/s00253-024-13354-5.

## Introduction

In nature, sulfated compounds play an important role in biology such as signal transduction, extracellular interaction, molecular recognition, and detoxification (Baba and Ishibashi 2019; Mikami and Kitagawa 2017; Papadopoulos et al. 2018). Consequently, they are very interesting targets due to their potential pharmacological applications. Furthermore, sulfated molecules are involved in the synthesis of a broad range of everyday chemical commodities like detergents, pigments, and agrochemicals (Takei et al. 1985; Tao et al. 2005). However, chemical sulfation methods generally entail the use of harsh chemical conditions such as the presence of highly reactive compounds, acidic conditions, organic solvents, or high temperatures (Al-Horani and Desai 2010). Moreover, they often display low chemo- and/or regioselectivity, as well as unwanted side reactions. Hence, these processes require additional protection/deprotection steps, which thereby reduce the overall yields and result in considerable amounts of waste (Al-Horani and Desai 2010; Simpson and Widlanski 2006). Alternatively, the development of enzyme-based strategies for sulfation of the desired compounds may entail some advantages, since these approaches could be performed under mild conditions and biocatalysts usually display high chemo- and regioselectivity (Bell et al. 2021; Hall 2021; Kissman et al. 2024; Sheldon et al. 2020; Winkler et al. 2021; Wu et al. 2021). For this purpose, sulfotransferases (STs) are the obvious choice as their natural role is to catalyze the transfer of a sulfate group from an activated donor to the hydroxy group of an acceptor (Chapman et al. 2004).

**Fig. 1:**
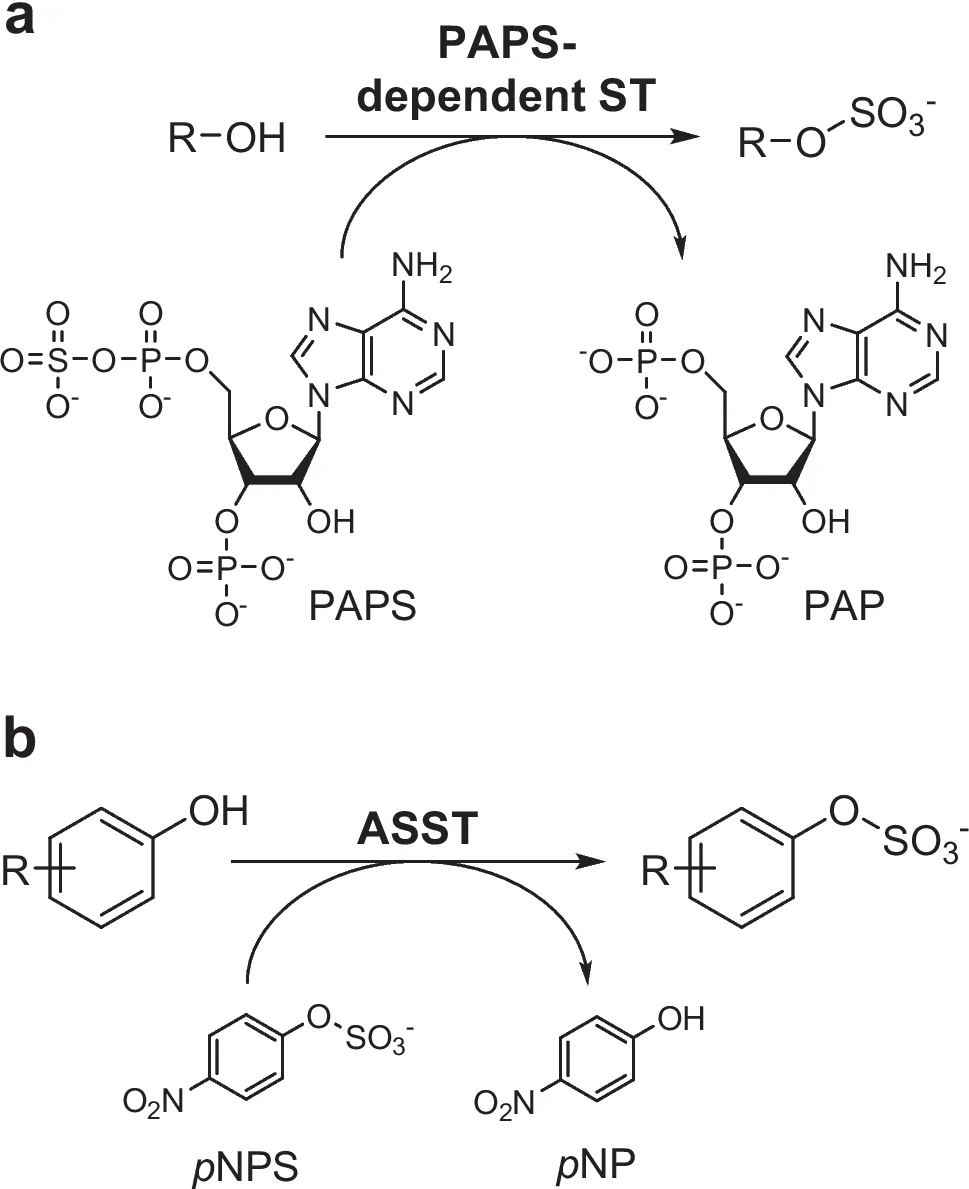
Sulfation reaction catalyzed by the two classes of sulfotransferase (ST). **a** PAPS-dependent ST and **b** PAPS-independent ST, i.e., ASSTs

Two classes of sulfotransferases can be distinguished according to their specificity towards the sulfate donor: 3′-phosphoadenosine-5′phosphosulfate (PAPS)-dependent and PAPS-independent sulfotransferases, also known as arylsulfate sulfotransferases (Bojarová and Williams 2008; Negishi et al. 2001) (Fig. 1). Concerning their potential synthetic applicability, PAPS-dependent sulfotransferases are well known for their regioselectivity and diverse substrate scope that includes the sulfation of proteins, lipids, and polysaccharides, as well as xenobiotics, hormones, and neurotransmitters (Badri et al. 2021; Falany 1997; James and Ambadapadi 2013; Lightning et al. 2021). However, their strict dependency for PAPS as an expensive sulfate donor, which is additionally unstable and causes enzyme inhibition, constitutes the PAPS-dependent STs’ main drawback and hampers their use as biocatalysts at large scale (Fig. 1A). To overcome this problem, efforts to create cofactor regeneration systems containing a second or more enzymes were developed (Ayuso-Fernández et al. 2014; Burkart et al. 2000, 1999; Lin et al. 1995; Monterrey et al. 2023). Nonetheless, these approaches add complexity to the biotransformation and require a tight control of the reaction conditions. ASSTs seem to allow a simpler handling since they are able to accept phenolic sulfate esters (e.g., *p*NPS or *p*-methylumbelliferyl sulfate (*p*MUS)) as sulfate donors (Fig. 1B) (Kobashi et al. 1987; Malojčić and Glockshuber 2010). These compounds are inexpensive compared to PAPS, making them more attractive from a synthetic point of view. Concerning their substrate scope, ASSTs preferentially transfer the sulfate moiety to phenolic alcohol groups from a wide range of hydroxy aromatic compounds such as antibiotics (Kaysser et al. 2010; Kim et al. 1992), steroids (van der Horst et al. 2012), or flavonoids (Purchartová et al. 2013; Valentová et al. 2017; van der Horst et al. 2015). Nonetheless, the sulfation of non-phenolic compounds, such as aliphatic alcohols and sugars, was also reported either by natural (Funabashi et al. 2010; Hartog and Wever 2015; Kaysser et al. 2010; van der Horst et al. 2012) or engineered ASSTs (Islam et al. 2018b; Koryakina et al. 2011), although at a slower rate.

Hence, taking into account the interesting possibilities that ASSTs offer in terms of sulfate acceptor, the goal of this work was to expand the biocatalytic toolbox to access a broad scope of sulfated compounds. For this purpose, our work focused on finding and characterizing new ASSTs (i.e., ASSTs from *Desulfitobacterium dehalogenans* (ASST*Ddeh*), *Dehalobacterium formicoaceticum* (ASST*Dfor*), *Desulfitobacterium hafniense* (ASSTC), *Hungatella effluvii* (ASST*Heff*), and *Desulfosporosinos orientis* (ASST*Dor*)), optimizing their reaction conditions and studying their acceptor substrate scope, including aromatic and aliphatic (primary and secondary) hydroxy and amino compounds for the aforementioned novel ASSTs, as well as the most interesting ones selected from the literature (i.e., ASSTA and ASSTB from *D. hafniense*) (Islam et al. 2018b; van der Horst et al. 2012).

## Materials and methods

### Materials

Tryptone and yeast extract were obtained from Oxoid, while sodium chloride was purchased from Carl Roth. Tris/HCl, sodium carbonate, 2-amino-2-methyl-1-propanol (AMP), imidazol, phenol (**1**), resorcinol (**3**), hydroquinone (**4**), 1,2,4-benzenetriol (**5**), 1,3,5-benzenetriol (**6**), 4,4′-dihydroxybiphenyl (**7**), 2-naphthol (**8**), aniline (**9**), uridine (**14**), *N*-acetylglucosamine (**15**), 1-hexanol (**16**), *rac*-1,5-hexanediol (**17**), *rac*-2-hexanol (**18**), *p*NPS, *p*NP, kanamycin, dimethyl sulfoxide (DMSO), 2-propanol, 1,2-dimethoxyethane [1,2-(MeO)_2_Et], and MOPS were bought from Sigma-Aldrich. Bis/Tris was ordered from Merck; sodium phosphate, catechol (**2**), and cyclohexanol (**10**) from Fluka; ethanol 96%, *N*,*N*-dimethylformamide (DMF), methanol, and isopropyl-β-d-thiogalactopyranoside (IPTG) from VWR; acetone from Acros Organics; and *all*-*rac*-cyclohexane-1,2-diol (**11**), *all*-*rac*-2-aminocyclohexanol (**12**), cyclohexylamine (**13**), and *all*-*rac*-2-methoxycyclohexanol (**19**) from BASF.

### Methods

#### Bioinformatic tools

BLASTp was run against the NCBI non-redundant (nr) database to find protein sequences displaying homology with ASSTA (Altschul et al. 1990, 1997, 2005) (Table S1). All parameters used were the ones defined as standard. Similarly, the protein homology tree was provided by NCBI taxonomy (Fig. S1). Sequence alignments between ASSTA from *D. hafniense* and the amino acid sequences retrieved from the BLAST search were performed using CLC Main Workbench 7.7 software (Fig. S2).

#### Plasmid design and cloning

Constructs enclosing the ASST genes were selected from literature (i.e., ASSTA (van der Horst et al. 2012), ASSTB (Islam et al. 2018b)) and the ASSTA-homologous enzymes (ASST*Heff*, ASST*Dor*, ASST*Dfor*, ASSTC, and ASST*Ddeh*) were designed as described in the Supporting Information (Table S2) and ordered from BioCat GmbH. All plasmids were transformed into *E. coli* BL21(DE3) and *E. coli* Neb5α for overexpression and storage purposes, respectively. Thus, chemo-competent cells stored at − 80 °C (see below) were thaw on ice. Subsequently, 80 µl of competent cell suspension was mixed with 1 ng of the corresponding plasmid and incubated on ice for 30 min. Afterwards, the mixture was subjected to heat shock (42 °C, 10 s) and 250 µl of LB was added. This was followed by incubation at 37 °C and 120 rpm for 2 h. Finally, 200 µl from each transformation was plated on an LB-agar plate containing kanamycin (50 µg/ml) and incubated at 37 °C O/N.

#### Preparation of competent cells

*Escherichia coli* chemo-competent cells were prepared by inoculating 50 ml of LB medium containing 0.5 ml MgSO_4_ (1 M) and 0.5 ml MgCl_2_ (1 M) with 1% of an O/Nc of the corresponding *E. coli* strain. The culture was then incubated at 37 °C and 120 rpm until OD_600_ ~ 0.7. Once reached the OD_600_, the culture was centrifuged at 4500 rpm and 4 °C for 15 min. The pellet was resuspended with a sterile ice-cold mixture of 4 ml of TMF buffer (CaCl_2_ 100 mM, RbCl_2_ 50 mM, and MnCl_2_ 40 mM) and 1 ml of 100% glycerol. The cells were then aliquoted, frozen with liquid nitrogen, and stored at − 80 °C until further use.

#### Overexpression and purification

Overexpression of the different ASSTs was performed using the following conditions (Table S2): a colony bearing the corresponding ASST plasmid was added to 10 ml LB medium containing kanamycin (50 µg/ml) and incubated at 37 °C and 120 rpm. Then, 500 ml cultures were inoculated with 1% volume using the corresponding *E. coli* O/N culture. After inoculation, cultures were incubated at 37 °C and 120 rpm until OD_600_ ~ 0.6. At that point, induction was started by adding IPTG to a final concentration between 0.4 and 1.0 mM and decreasing the temperature to 20 °C or 30 °C O/N, depending on the ASST. The cultures were centrifuged at 4 °C and 4500 rpm for 15 min. The supernatant (SN) was discarded, and the cell pellet was resuspended in 40 ml of buffer Tris/HCl 50 mM, at pH 8.0. Cell lysis was performed by ultrasonication (Digital Sonifier, Branson) at 30% amplitude for 5 min (1 s pulse on, 5 s pulse off) on ice. The suspension was centrifuged at 4500 rpm, 4 °C for 15 min. Subsequently, the supernatant, containing the cell-free extract (CFE), was aliquoted and snap frozen with liquid nitrogen. The frozen samples were stored at − 20 °C until further usage.

Protein purification was achieved by metal affinity chromatography. CFE of the corresponding ASST (5 ml) was thawed on ice and purified on a HisTrap™ FF (5 ml) using an AKTA-FPLC system (GE Healthcare Life Science). The column was pre-equilibrated with binding buffer (50 mM sodium phosphate buffer at pH 7.5). After injection of the CFE (0.5 ml/min), the column was washed with 5 column volumes of binding buffer at 3 ml/min. Subsequently, the corresponding ASST was eluted with the same buffer but containing in addition 0.25 M imidazole. All the fractions with ASST were pooled together, desalted by PD10 columns (GE Healthcare), aliquoted, and frozen in liquid nitrogen. The frozen samples were stored at − 20 °C until further usage.

#### Determination of protein concentration

Protein concentration was determined by the bicinchoninic acid (BCA) assay (Pierce™ BCA Protein Assay Kit, Thermo Fisher Scientific, Waltham, USA) with bovine serum albumin as a standard. The absorbance of all samples was measured in triplicates (*λ* = 562 nm) (SpectraMax M2, Molecular Devices). Alternatively, absorbance at 280 nm of the corresponding enzyme solution was measured (IMPLEN NanoPhotometer NP80), and the protein concentration was calculated using the ASST molecular weight and theoretical molar extinction coefficient (Table S3).

#### SDS-PAGE

Sodium dodecyl sulfate polyacrylamide gel electrophoresis (SDS-PAGE) was performed using precast ExpressPlus™ PAGE gels (10% acrylamide) purchased from GenScript. Gels were run on a Mini-PROTEAN Tetra System–Mark apparatus (Bio-Rad) at 100 V. Molecular weight marker was purchased from Thermo Scientific (Prestained protein ladder, 10–180 kDa). Proteins were stained with Coomassie Brilliant Blue G-250 (Bradford).

#### Calculation of the *p*-nitrophenol molar extinction coefficient

The corresponding *p*NP molar extinction coefficient was calculated by measuring spectrophotometrically (SpectraMax M2, Molecular Devices, or FLUOstar Omega, BMG) the absorbance at increasing concentrations of *p*NP (0.0 to 0.25 mM) in the different reaction conditions employed to determine the ASST activity. For this purpose, measurements were done in triplicates at two different wavelengths: *λ* = 410 nm (maximum absorption wavelength) and/or *λ* = 350 nm (*p*NP isosbestic point) (Table S4).

#### Standard conditions for the activity measurement of the ASSTs

Unless stated otherwise, sulfotransferase activity was determined by following the initial hydrolysis rate of *p*NPS spectrophotometrically during 30 min (SpectraMax M2, Molecular Devices, or FLUOstar Omega, BMG) at *λ* = 410 nm in a 96-well microtiter plate. The assays were performed at 30 °C in 200 µl buffer (Tris/HCl 50 mM, pH 8.0) containing *p*NPS (1 mM), phenol (1 mM), and the desired ASST (in CFE or purified), i.e., ASSTA, ASSTB, ASST*Heff*, ASST*Dor*, ASST*Dfor*, ASSTC, or ASST*Ddeh*. Reaction blanks were performed by adding all the mixture components with the exception of the acceptor substrate. All measurements were performed at least in triplicates.

One unit of arylsulfate sulfotransferase activity was defined as the amount of enzyme releasing 1 µmol of *p*NP per minute.

#### Study of the reaction conditions for the novel ASSTs

##### Optimal reaction temperature

Optimal reaction temperature was determined by measuring the activity of the corresponding purified ASSTA-homologs at temperatures ranging from 25 to 45 °C. Reaction composition was prepared as previously described.

##### Optimal pH

The activity of the ASSTA homologs was measured at different pH values. Reactions were performed under standard reaction conditions, with the exception of the buffer present. The pH was adjusted using the following buffers (50 mM): Bis/Tris (pH 5.5, pH 6.0, and pH 7.0), sodium phosphate (pH 7.0, pH 7.5, and pH 8.0), Tris/HCl (pH 8.0, pH 8.5, and pH 9.0), sodium carbonate (pH 9.0, pH 10.0, and pH 10.5), or AMP (pH 9.0, pH 10.0, and pH 10.5). Sulfotransferase activity was determined by following the initial hydrolysis rate of *p*NPS spectrophotometrically for 30 min (SpectraMax M2, Molecular Devices) at *λ* = 410 nm or *λ* = 350 nm, depending on the pH. In order to calculate the ASST activity at each pH, the corresponding molar extinction coefficient was determined as previously described (Table S4).

##### Co-solvent tolerance

Sulfotransferase activity of the ASSTA homologs was analyzed in different mixtures of aqueous buffer plus 10% (v/v) organic co-solvent. Reactions were carried out in the standard conditions described above. The assayed co-solvents were 2-propanol, methanol, acetone, DMF, 1,2-(MeO)_2_Et, and dimethyl sulfoxide (DMSO). In order to calculate the ASST activity in the presence of each co-solvent, the corresponding molar extinction coefficient was previously determined (see section “Calculation of the *p*-nitrophenol molar extinction coefficient”) (Table S4).

#### ASST activity measurement towards different acceptor substrates

Sulfotransferase activity was determined by following the initial rate of *p*NP formation from *p*NPS spectrophotometrically for 30 min (SpectraMax M2, Molecular Devices) at *λ* = 410 nm in a 96-well microtiter plate. The assays were performed under standard conditions except for the concentration of the acceptor substrate. As acceptor substrate (1, 10, or 100 mM), the following substrates were tested: phenol (**1**), catechol (**2**), resorcinol (**3**), hydroquinone (**4**), 1,2,4-benzenetriol (**5**), 1,3,5-benzenetriol (**6**), 4,4′-dihydroxybiphenyl (**7**), 2-naphthol (**8**), aniline (**9**), cyclohexanol (**10**), *all*-*rac*-cyclohexane-1,2-diol (**11**), *all*-*rac*-2-aminocyclohexanol (**12**), cyclohexylamine (**13**), uridine (**14**), *N*-acetylglucosamine (**15**), 1-hexanol (**16**), *rac*-1,5-hexanediol (**17**), *rac*-2-hexanol (**18**), and *all*-*rac*-2-methoxycyclohexanol (**19**). Reactions containing 4,4′-dihydroxybiphenyl (10 mM and 100 mM) could not be performed due to the poor water solubility of the substrate at the aforementioned concentrations. Reaction blanks were also performed by adding all the mixture components except for the acceptor substrate. All measurements were performed at least in triplicates.

One unit of arylsulfate sulfotransferase activity was defined as the amount of enzyme releasing 1 µmol of *p*NP per minute.

#### Determination of the kinetic parameters

Enzyme kinetic parameters of the ASSTs were determined for *p*NPS (0–20 mM) employing the standard assay with acceptor substrate (constant, 100 mM) and the corresponding purified ASST (50 nM). These parameters were investigated using both phenol (**1**) and catechol (**2**) as sulfate acceptor. All kinetic studies were prepared and measured in an automated manner at our robotic platform Xavier (Fig. S3). Reactions were performed in triplicates. Initial velocities (*V*_i_) were fitted to the Michaelis–Menten equation, and kinetic constants (*V*_max_ and *K*_M_) were obtained using the built-in non-linear regression tools in SigmaPlot 15.0. Values presented are the mean of three replicate measurements.

#### Melting temperature

The melting temperature of the ASSTs was measured using the Thermofluor assay using a BioRad® CFX Connect Real time PCR system (BioRad, Hercules, CA, USA). The experiments were carried out in 96-well plates by adding Sypro® Orange as fluorescent dye (2.5 µl in 50 mM MES buffer pH 7.0) to the corresponding ASSTs (1 mg/ml final conc.) dissolved in buffer (Tris/HCl 50 mM, 25 µl final volume). Each enzyme melting temperature was determined at two different pHs, 8.0 and 9.0, in triplicates. The starting temperature of 20 °C was kept for 5 min, then the temperature was increased at a rate of 0.5 °C/min to 95 °C. Melting temperatures were determined using the program BioRad CFX Manager 3.0.

#### Disulfation of di- or trihydroxy aromatic acceptors

The capability of transferring more than one sulfate group to catechol (**2**), resorcinol (**3**), hydroquinone (**4**), 1,2,4-benzenetriol (**5**), 1,3,5-benzenetriol (**6**), and 4,4′-dihydroxybiphenyl (**7**) was tested in two sets of reactions with varying reaction time and substrate concentration.

The first set of reactions was carried out in 1,4-piperazinediethanesulfonic acid buffer (PIPES 20 mM pH 8.0, 100 µl final reaction volume), containing 1.5 mM of *p*NPS and 0.5 mM of acceptor substrate. Reactions were started by adding the corresponding purified ASSTs (30 µl; ASSTA, 0.06 mg/ml; ASSTB, 0.17 mg/ml; ASST*Heff*, 0.57 mg/ml; ASST*Dor*, 0.16 mg/ml; ASST*Dfor*, 0.42 mg/ml; ASSTC, 0.50 mg/ml; ASST*Ddeh*, 0.45 mg/ml final reaction concentration). Reaction progress was followed spectrophotometrically at 410 nm until no further increase of absorbance was measured (final reaction time from 30 min to 3 h). Then, reactions were quenched by addition of 100 µl of acetonitrile. Product formation of all ASSTs (i.e., ASSTA, ASSTB, ASST*Heff*, ASST*Dor*, ASST*Dfor*, ASSTC, or ASST*Ddeh*) was analyzed in this reaction set.

The second set of reactions was performed using similar conditions as before, although with the following differences: (i) 500 µl final volume reactions in 1.5 ml microtubes; (ii) substrate concentrations for the donor and acceptor were 30 mM and 10 mM, respectively; (iii) reactions were shaken at 650 rpm during 16 h without following *p*NP formation spectrophotometrically; and (iv) only ASSTB, ASSTC, and ASST*Ddeh* were added as biocatalysts (50–150 ng/ml of reaction).

Both reaction sets were performed at 30 °C, stopped with one reaction volume of acetonitrile. The quenched reaction mixtures were centrifuged either for 5 min at 4500 rpm (reactions performed in MTPs) or during 2 min at 14,000 rpm (reactions performed in microtubes), and the supernatant was transferred into vials.

The first set of reactions was analyzed by flow injection mass spectrometry (FIA-MS). For this purpose, samples were injected into an Agilent 1260 Infinity HPLC system (G1311B quaternary pump, G1329B autosampler, G1316A thermostated column compartment, G1314F variable wavelength detector) equipped with a *Phenomenex* SecurityGuard™ C18 cartridge and coupled to an Agilent 6120 single-quadrupole MS detector (Berger et al. 2023). Using the Agilent LC/MS ChemStation B.04.03 software, the specific substrate and product ion chromatograms were extracted from the MS data measured in scan-mode (m/z, 30–500) (Table S5 and Table S6).

The second set of reactions was analyzed by HPLC–MS were injected on an *Agilent*1260 Infinity HPLC system equipped with a *Phenomenex* Luna C18 column (dimensions, 250 mm × 4.6 mm; stationary phase, fully porous silica, C18 with TMS end capping; particle size, 5 µm; pore size, 100 Å) preceded by a *Phenomenex* SecurityGuard™ C18 cartridge and coupled to an *Agilent*6120 single-quadrupole MS detector. The method used is described in Table S7.

## Results

### Identification and overexpression

In order to identify new natural ASSTs with possible complementary catalytic characteristics, an in silico screening was performed first. The previously described enzyme ASSTA was used as template in a pBLAST search, which retrieved 29 different DNA sequences displaying a sequence identity between 93 and 55% (Table S1). A dendrogram presenting the different clusters of sequences according to their distances from the query sequence revealed four main branches (Fig. S1). To cover the whole range of structural diversity displayed by these sequences in wet-lab experiments, one or two proteins from each branch were chosen: (i) ASST from *Desulfitobacterium dehalogenans* (93.2% homology); (ii) ASST from *Dehalobacterium formicoaceticum* (86.8% homology); (iii) an unprecedent ASST from *Desulfitobacterium hafniense* (71.2% homology); (iv) ASST from *Hungatella effluvii* (58.5% homology); and (v) ASST from *Desulfosporosinos orientis* (58.8% homology). With the exception of ASST*Ddeh*, whose sequence showed the highest homology with ASSTA, the selection of the other enzymes was based on the degree of dissimilarity presented in the conserved region around the proposed catalytic histidine (H351) (van der Horst et al. 2012) (Fig. S2). This criterion was followed because it can be safely assumed that these amino acids form part of (or are nearby) the active site, and their variation could have a higher impact on the protein substrate specificity. Hence, the study of their preferences for the sulfation of different acceptor compounds could give hints about key positions and substitutions to modulate or enhance the acceptance of molecules not bearing aromatic alcohols.

Plasmids including each of the selected genes were designed, purchased, and transformed into *E. coli* BL21 (DE3) cells (Table S2). After growing and disrupting the *E. coli* cells, protein overexpression was verified by sodium dodecyl sulfate–polyacrylamide gel electrophoresis (SDS–PAGE) analysis. Strong protein bands (Fig. S4) matching the expected molecular weight (⁓70–76 kDa, Table S3) were observed in the cell-free extract (CFE) fraction from all the corresponding ASST cultures. Hence, all of them could be overexpressed in soluble form, although the overexpression levels varied depending on the enzyme. The presence of the ASSTs as inclusion bodies was observed at varied level except for the enzyme from *H. effluvii*. Purification of the overexpressed ASSTs was easily achieved by IMAC chromatography thanks to the presence of a His-tag at the C-terminus of each protein (Fig. S5 and Table S2).

A preliminary assay to confirm the sulfate transferase activity of the ASSTA-homologs was performed using *p*NPS as sulfate donor. For this purpose, the activity of each of the ASST-containing CFEs was measured towards four different acceptor substrates namely phenol, catechol, uridine, and *N*-acetylglucosamine (GlcNAc) (Table S8). At this stage, only the activity towards phenol and catechol could be detected. Similarly as observed before for other ASSTs described in the literature, in all the cases, the activity towards catechol was higher than towards phenol (Ayuso-Fernández et al. 2014), ranging between 2–135 and 0.1–22 U/ml_CFE_, respectively. Concerning the lack of product formation in the uridine and GlcNAc reactions, as mentioned below, this could be due to the necessity of higher concentrations of acceptor substrate and/or enzyme in the reaction (van der Horst et al. 2012).

### Functional characterization

The ASSTs were characterized with respect to optimal temperature, pH, their tolerance towards the presence of co-solvents in the reaction medium, and their kinetic parameters.

#### Optimal reaction temperature and pH

**Fig. 2:**
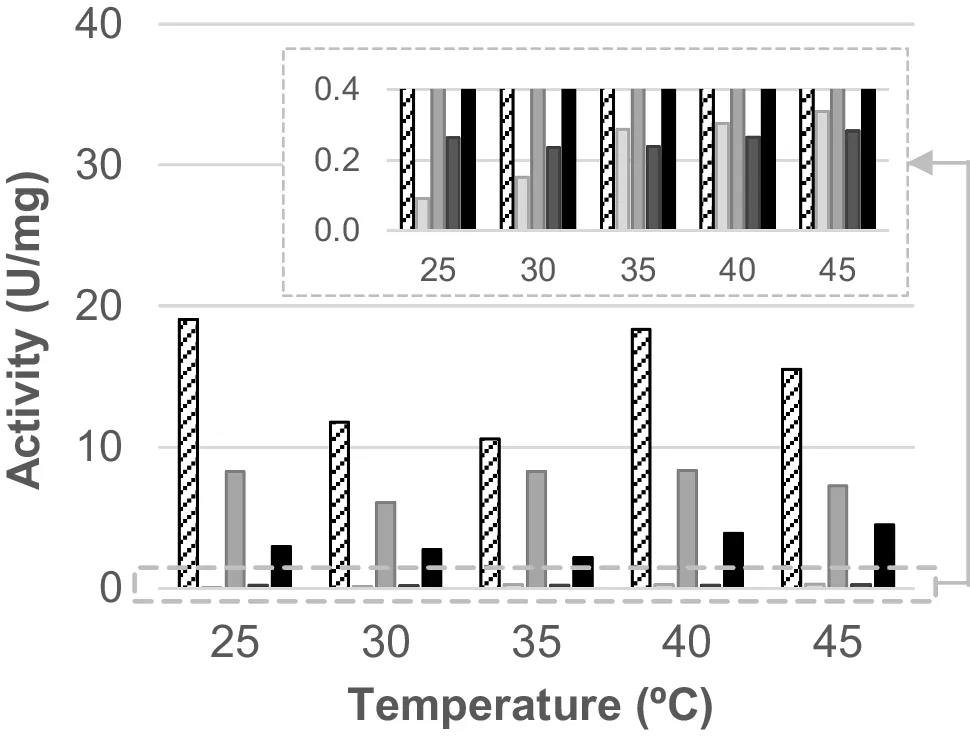
Specific activity displayed by ASST*Heff* (white box), ASST*Dor* (light gray box), ASST*Dfor* (gray box), ASSTC (dark gray box), and ASST*Ddeh* (black box) depending on the reaction temperature. The insert shows an amplification of the plot between 0.0 and 0.4 U/mg of protein to facilitate the visualization of the activity from the less active ASSTs. Reactions were carried out in buffer Tris/HCl 50 mM, pH 8.0; phenol 1.0 mM; *p*NPS 1.0 mM; and purified ASST. Kinetic measures were followed during 30 min at temperatures between 25 and 45 °C

To determine their optimal temperature, the activity per mg of purified ASST was measured at temperatures between 25 and 45 °C using phenol and *p*NPS as substrates. All the ASSTs were active in the tested temperature range, although clear differences in terms of absolute activity were observed (Fig. 2). The enzyme ASST*Heff* displayed the highest activity at all temperatures (between 10 and 19 U/mg of protein), followed by ASST*Dfor* (6–8 U/mg of protein) and ASST*Ddeh* (3–4 U/mg of protein), while ASSTC and ASST*Dor* displayed significantly lower activity (between 0.2–0.3 and 0.1–0.3 U/mg of protein, respectively). Concerning the optimal temperature, most ASSTs exhibited the best activity values at the higher temperatures, i.e., either 40 °C (ASST*Dfor*) or 45 °C (ASST*Dor*, ASSTC, and ASST*Ddeh*), while ASST*Heff* displayed maximum activity at 25 °C.

**Fig. 3:**
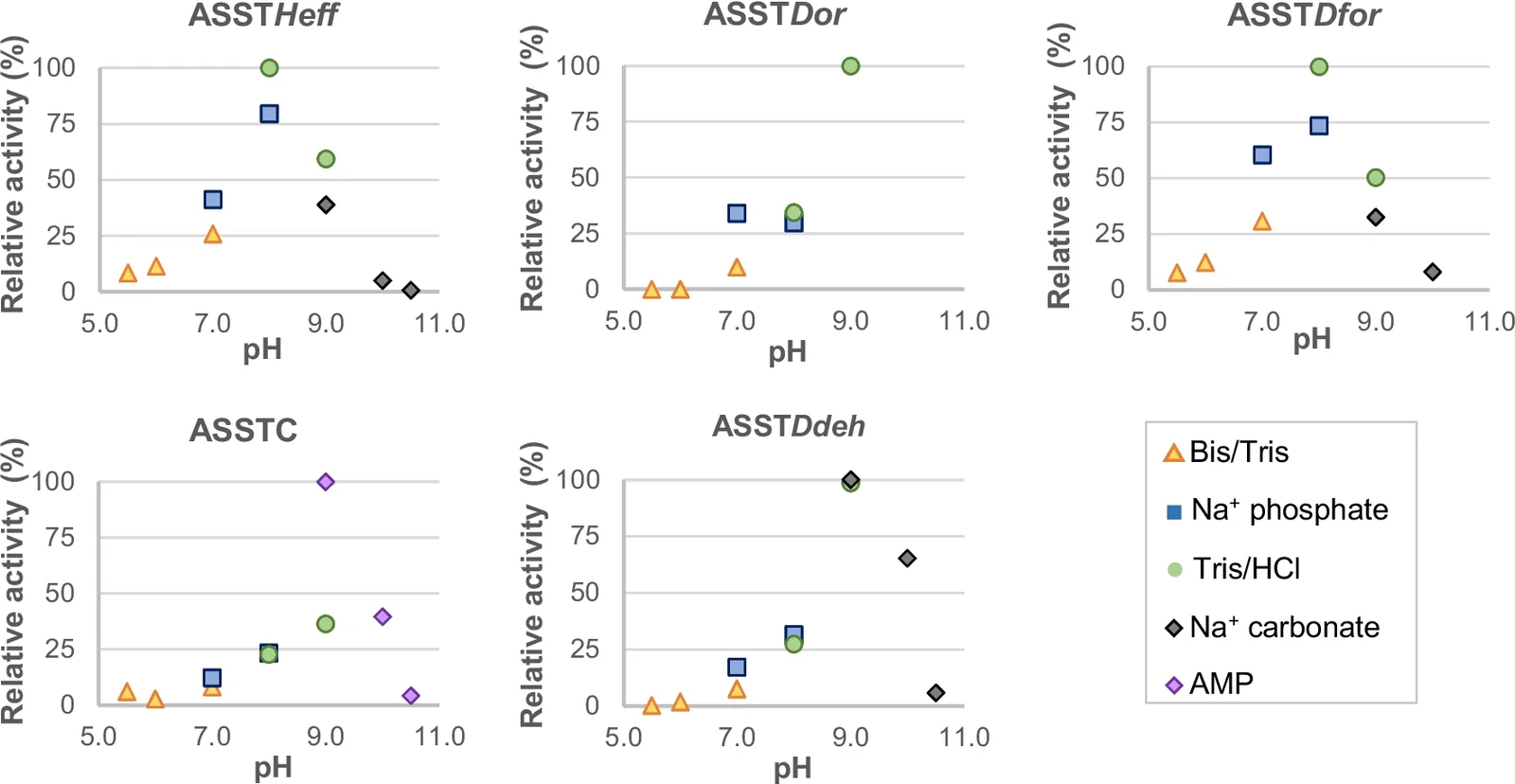
Relative activity displayed by ASST*Heff*, ASST*Dor*, ASST*Dfor*, ASSTC, ASST*Ddeh* depending on the reaction pH. Reactions were carried out with phenol 1.0 mM, *p*NPS 1.0 mM, and purified ASST. Kinetic measures were followed at 30 °C during 30 min. Reactions with ASST*Dor* were not carried out at pH above 9.0 due to precipitation of the enzyme in the reaction

In a similar experiment, the optimal pH of the novel ASSTs was determined by measuring the rate of *p*NP formation at varied pH values (Fig. 3). The values obtained showed a sharp preference of the ASSTs for pH 8.0 (ASST*Heff* and ASST*Dfor*) or 9.0 (ASST*Dor*, ASSTC, and ASST*Ddeh*). Especially not well tolerated were pHs below 7.0, even causing the precipitation of ASSTC (pH 5.5 and 6.0) and ASST*Ddeh* (pH 5.5). These optimal pH values are in the range to those described for ASSTA (pH 9.5) (van der Horst et al. 2012) and other arylsulfate sulfotransferases (Hossain et al. 2011; Islam et al. 2018b; Kim et al. 1992).

#### Co-solvent tolerance

**Fig. 4:**
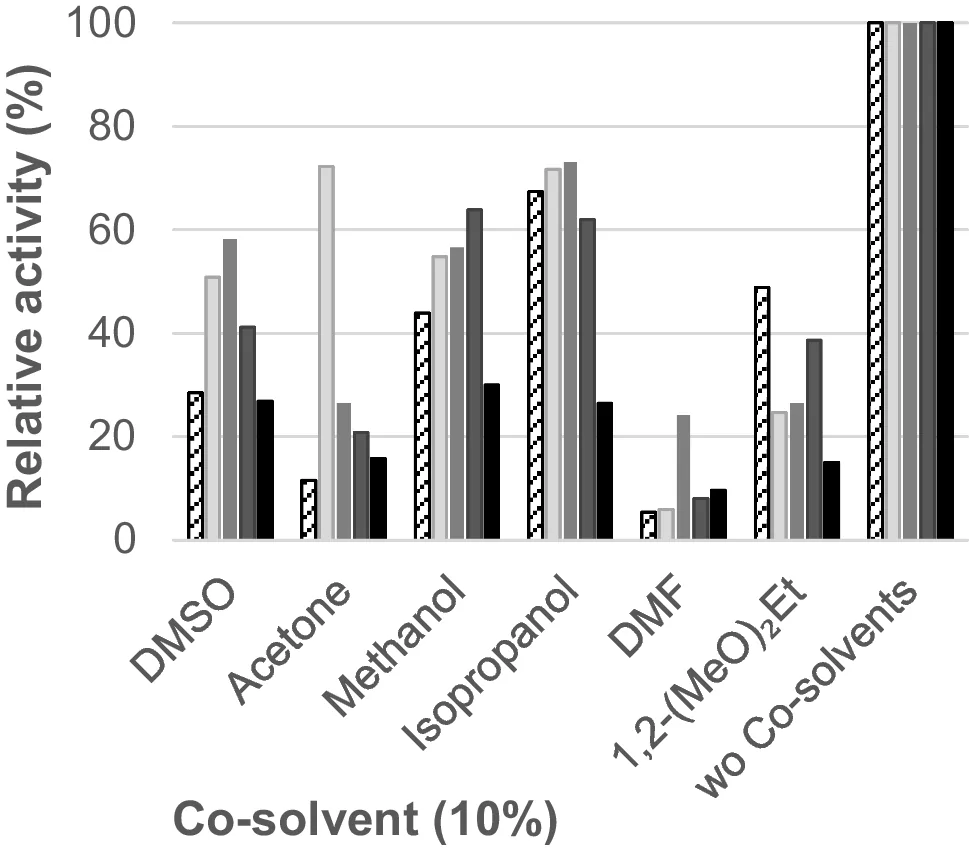
Relative activity displayed by ASST*Heff* (white box), ASST*Dor* (light gray box), ASST*Dfor* (gray box), ASSTC (dark gray box), ASST*Ddeh* (black box) depending on the presence of co-solvents (10% v/v). Reactions were carried out in buffer Tris/HCl 50 mM, pH 8.0 with 10% (v/v) DMSO, phenol 1.0 mM, *p*NPS 1.0 mM, and purified ASST. Kinetic measures were followed during 30 min at temperatures at 30 °C

Numerous compounds that are interesting candidates for sulfation show very low solubility in water. Poor solubility of the substrates in the reaction media entails low substrate availability for the enzyme, which translates in less efficient product formation. Thus, the use of organic co-solvents represents one possible solution to overcome this problem. A study of the tolerance of the novel ASSTs towards the presence of different co-solvents was carried out. For this purpose, the ASST activity was monitored in reactions containing 10% (v/v) of organic solvents commonly used in biocatalysis such as DMSO, acetone, methanol, 2-propanol, DMF, and 1,2-(MeO)_2_Et (Fig. 4). The presence of organic solvents affected the enzyme activities to different extents. In general, the ASSTs retained high percentages of their activities in the presence of 2-propanol, methanol, and DMSO. In the presence of these co-solvents (10% v/v), most enzymes displayed around 60, 50, and 40% of their initial activity, for each co-solvent, respectively. On the contrary, DMF showed to be the less tolerated organic solvent. In this case, ASST activity was in general below 10%, only ASST*Dfor* kept around 25% of its activity. It is noteworthy that, although acetone and 1,2-(MeO)_2_Et were generally not among the best tolerated organic solvents, they were reasonably tolerated in selected cases like ASST*Dor* (72%) and ASST*Heff* (49%). Concerning the overall behavior of the enzymes, the results indicate that ASST*Dor* and ASST*Dfor* were the most stable enzymes in presence of organic co-solvents, while ASST*Ddeh* exhibited the lowest tolerance. The latter enzyme lost approximately 80% of its activity regardless of the solvent added to the reaction.

#### Melting temperature

The melting temperature (Tm) of a protein is defined as the temperature at which the concentration of the protein in its folded state equals the concentration of the unfolded protein under certain conditions. This parameter is a common descriptor to quantify the thermal stability of proteins and has a wide range of potential applications from drug design to the optimization of enzyme activity. To determine this value for the ASSTs, a thermal shift assay (also known as differential scanning fluorimetry or Thermofluor assay) was performed. This assay follows protein denaturation in presence of an added environment-sensitive fluorescent dye by monitoring the fluorescence increase upon thermal protein unfolding, caused by interaction of the dye with the exposed hydrophobic core of the denatured protein (Pantoliano et al. 2001). This experiment was performed at pH 8.0 and 9.0, since they were the best tolerated pH values. In addition to the newly characterized ASSTs, ASSTA and ASSTB were also included. The melting temperature ranged between 38 and 45 °C at pH 8.0 and 36 and 40 °C at pH 9.0 (Table S9). Independently on the catalytic optimal pH of the enzyme, the melting temperature was higher at pH 8.0. The only exception was ASST*Dor*, which displayed a melting temperature of 38 °C at both pHs. It is worth to note that the melting temperature did not correlate with the solvent stability, since ASST*Ddeh* displayed a higher melting temperature (39.0 °C) than ASST*Dor* (38.0 °C), but ASST*Ddeh* was clearly less solvent tolerant (Fig. 4).

### Kinetic parameters

For determining the kinetic parameters (*V*_max_; Michaelis–Menten constant, *K*_M_; *k*_cat_; and catalytic efficiency, *k*_cat_/*K*_M_), the initial activities were measured spectrophotometrically based on the *p*NP formation rate at varying sulfate donor concentrations (i.e., *p*NPS, 0–20 mM) and constant acceptor concentration (100 mM). Additionally, in order to evaluate a possible influence of the acceptor substrate on the kinetic parameters for the donor substrate, phenol and catechol were used as sulfate acceptor.

All the ASSTs displayed a Michaelis–Menten behavior towards *p*NPS (Fig. S6 and Fig. S7). Depending on the acceptor substrate (phenol or catechol), the values varied (Table 1). The highest reaction rates were achieved in all cases with catechol (**2**) as second substrate, whereby the difference between phenol and catechol depended on the enzyme. For instance, the *V*_max_ was quite similar with both substrates for ASSTB, while it was approximately 25 times faster for catechol with ASST*Dfor*. The acceptor substrate also influenced the *K*_M_ values, although there was no general trend shared by all the enzymes. For instance, a higher affinity for the donor substrate was observed with phenol (**1**) for ASSTA, ASST*Heff*, ASST*Dfor*, ASSTC, and ASST*Ddeh*. Contrarily, ASSTB and ASST*Dor* showed better (lower) *K*_M_ values when catechol (**2**) was the acceptor substrate. Concerning the turnover number (*k*_cat_), the catalytic rate constants for each substrate ranged within an order of magnitude among the ASSTs, whereby the higher *k*_cat_ values were always found with catechol (from 0.6 to 61.2 s^−1^ and from 11.9 to 310.7 s^−1^ with phenol (**1**) and catechol (**2**), respectively). Once more, the highest catalytic efficiency studied for *p*NPS was detected in presence of catechol (**2**) as acceptor substrate for all the enzymes. The dissimilarity in the catalytic efficiencies related to the acceptor substrate was especially emphasized in the case of ASST*Dor* (aprox. 40 times higher *k*_cat_/*K*_M_). In contrast, both values were comparable for ASST*Heff* (22.3 and 33.1 mM^−1^ s^−1^ with phenol (**1**) and catechol (**2**), respectively). When collating the results gathered among the ASSTs, the highest substrate affinity was displayed by ASSTC and ASSTA (0.2 and 0.3 mM, respectively with phenol (**1**)) and by ASST*Dfor* and ASST*Ddeh* (0.5 mM for both with catechol (**2**)). In addition, ASSTA and ASST*Ddeh* also showed the highest catalytic efficiency (83.9 and 461.3 mM) in the presence of phenol (**1**) and catechol (**2**), respectively.

**Table 1:** Apparent kinetic parameters of ASSTA, ASSTB, ASST*Heff*, ASST*Dor*, ASST*Dfor*, ASSTC, and ASST*Ddeh* for *p*NPS at a fixed concentration of phenol (**1**) or catechol (**2**)^[a]^

Enzyme	Acceptor	*V*_max_ (µmol min^−1^ mg^−1^)	*K*_M_ (mM)	*k*_cat_ (s^−1^)	*k*_cat_* k*_M_^−1^ (mM^−1^ s^−1^)
ASSTA	**1**	21.2	0.3	26.9	83.9
	**2**	102.2	0.6	129.5	215.8
ASSTB	**1**	49.6	1.4	61.2	43.7
	**2**	57.0	0.6	70.3	117.2
ASST*Heff*	**1**	39.8	2.1	46.9	22.3
	**2**	263.7	9.4	310.7	33.1
ASST*Dor*	**1**	0.5	1.0	0.6	0.6
	**2**	10.1	0.5	11.9	23.7
ASST*Dfor*	**1**	5.3	0.5	6.3	12.6
	**2**	130.4	0.9	155.2	172.4
ASSTC	**1**	0.6	0.2	0.7	3.6
	**2**	11.8	1.6	14.0	8.7
ASST*Ddeh*	**1**	21.2	0.4	25.3	63.2
	**2**	193.3	0.5	230.7	461.3

^[a]^The corresponding Michaelis–Menten plots can be found in the supporting information Fig. S6 and Fig. S7. Reaction conditions: Tris/HCl buffer (50 mM, pH 8.0), *p*NPS (0–20 mM), acceptor substrate (100 mM, phenol (**1**), or catechol (**2**)) and the purified ASST (50 nM). Kinetic measures were followed during 30 min at 30 °C temperature

### Sulfate acceptor substrate scope

**Fig. 5:**
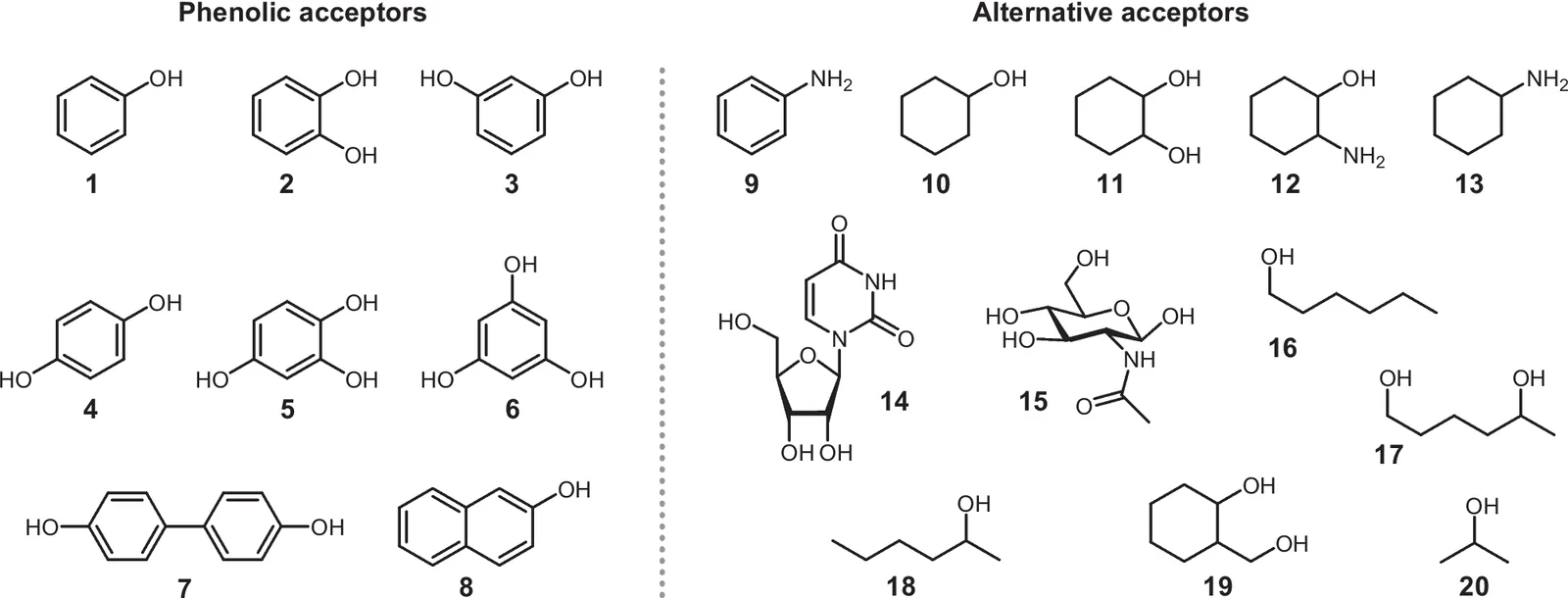
Compounds screened as sulfate acceptors for the ASSTs, i.e., phenol (**1**), catechol (**2**), resorcinol (**3**), hydroquinone (**4**), 1,2,4-benzenetriol (**5**), 1,3,5-benzenetriol (**6**), 4,4′-dihydroxybiphenyl (**7**), 2-naphthol (**8**), aniline (**9**), cyclohexanol (**10**), *all-rac*-cyclohexanediol (**11**), *all-rac*-2-aminocyclohexanol (**12**), cyclohexylamine (**13**), uridine (**14**), *N*-acetylglucosamine (**15**), 1-hexanol (**16**), *rac*-1,5-hexanediol (**17**), *rac*-2-hexanol (**18**), and *all-rac*-2-methoxycyclohexanol (**19**)

To study the acceptor substrate scope of the newly characterized ASSTs, as well as ASSTA and ASSTB, the activity of each of the enzymes was tested towards structurally diverse potential sulfate acceptors. The substrates used were grouped into phenolic (**1**–**8**) and alternative acceptors (**9**–**19**), comprising aniline (**9**) and aliphatic alcohols and amines (Fig. 5). Three acceptor concentrations were tested (1, 10, and 100 mM) in order to detect any effect of this parameter to the activity of the enzymes. The possible unspecific hydrolysis of *p*NPS was taken into account by setting blank reactions in the absence of acceptor substrate. Additionally, as 2-propanol was required in some cases as co-solvent to improve the solubility of the substrates, blank reactions were also run in the presence of 2-propanol (10% v/v). Interestingly, under these conditions, all ASSTs showed low, albeit significant, activity towards the sulfation of 2-propanol (max. 16 mU/mg, Fig. S8). In order to accurately calculate the activity of the ASSTs, the increase in absorbance during the kinetic measurements due to either of the aforementioned reasons was considered.

#### Activity towards phenolic compounds

**Fig. 6:**
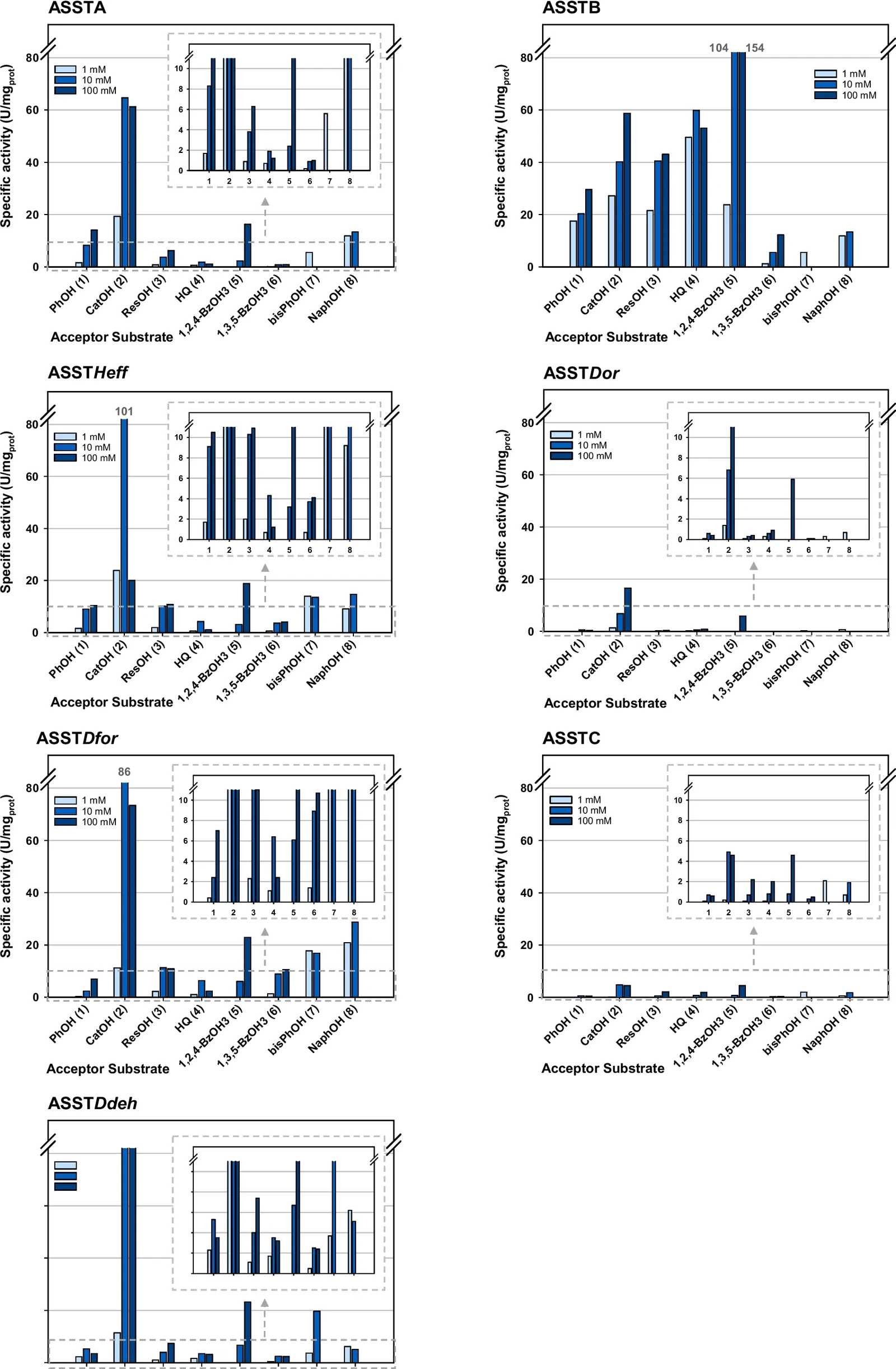
Specific activity of ASSTs towards phenolic compounds (**1**–**8**) and *p*NPS determined by colorimetric assay following *p*NP formation at 410 nm. Reactions conditions: Tris/HCl buffer (50 mM, pH 8.0), *p*NPS (1 mM), acceptor substrate (1, 10 or 100 mM), and the purified ASST

The specific activity towards eight phenolic compounds (**1**–**8**) was measured, and activity was observed for all substrates and all ASSTs studied reaching up to 154 U/mg (Fig. 6). Nevertheless, each enzyme displayed very distinctive substrate preferences and sulfation rates depending on the acceptor structure and its concentration. In most of the cases, activities were higher at elevated substrate concentrations.

Another general feature was that for all enzymes, the highest activities were measured with catechol (**2**) and 1,2,4-benzenetriol (**6**), although not necessarily in that order. An exception was ASST*Dfor*, which preferred 2-naphthol (**8**) over **6** as second-best acceptor substrate. The size of the aromatic alcohol did not lead to a common trend on the activity of the ASSTs. For instance, the bulkier substrates such as 4,4′-dihydroxybiphenyl (**7**) or 2-naphtol (**8**) were accepted by all ASSTs and actually preferred over some of the smaller acceptors (i.e., resorcinol (**3**), hydroquinone (**4**), or 1,3,5-benzenetriol (**6**)) by ASSTA, ASST*Heff*, and ASST*Dfor*.

The most substrate selective among all the studied enzymes was ASST*Dor*. This ASST exhibited an activity tenfold or higher for catechol (**2**, 100 mM 16.6 U/mg) than with the other phenolic substrates, except the second-best substrate, 1,2,4-benzenetriol (**5**, 100 mM, 5.9 U/mg). Concerning the absolute activity values displayed by each enzyme, the highest specific activities were displayed by ASSTB reaching up to 154 U/mg for **5** (lowest value 1.3 U/mg), while ASSTC showed the lowest activity values from 0.1 U/mg to still impressive 5 U/mg.

#### Disulfation of aromatic polyols

The phenolic set of substrates chosen can be subdivided into mono- or polyols (di- or trihydroxy compounds). Hence, it would be possible for the polyhydroxylated molecules to undergo several sulfate transfer steps. Using the colorimetric assay, no information can be obtained about the numbers of sulfate groups transferred. Consequently, a screening with the substrates **2**–**7** was carried out in 96-well microtiter plates. These reactions were performed in buffer PIPES (20 mM, pH 8.0), containing three times higher concentration of *p*NPS (1.5 mM) than the acceptor substrate (0.5 mM). Formation of *p*NP was followed spectrophotometrically (410 nm), and sulfate transfer was deemed finished when no further increase in absorbance was observed. To ensure reaching the end point before 3 h, a sufficient amount of biocatalyst was added to the reaction mixture. After stopping the biotransformations with acetonitrile, samples were analyzed by flow-injection mass spectrometry (FIA-MS). Due to the lack of (commercial) availability of the expected mono-, di-, and trisulfated products, the buffer was used as internal standard, and the obtained data was converted into target/buffer integral ratios (Berger et al. 2023). This method allowed to generate qualitative data about the sulfation pattern of each of the ASSTs and its comparison among the enzymes.

The expected formation of monosulfated compounds was detected by FIA-MS for all the ASSTs with catechol (**2**), resorcinol (**3**), hydroquinone (**4**), and 4,4’-biphenol (**7**). Unfortunately, the detection of the trihydroxybenzenes (**5** and **6**) and their derivatives was more challenging. For instance, the detection of 1,2,4-benzenetriol (**5**) or of its sulfated derivatives was not possible using this analytical method. Similarly, 1,3,5-benzenetriol (**6**) or its trisulfated counterpart was not detected, although low target/buffer integral ratios were identified for the monosulfated (and/or disulfated) compound with all the enzymes, but ASSTC (Fig. S9 and Fig. S10).

Concerning the ability of the enzymes to transfer more than one sulfate group onto the same substrate, disulfation of 4,4′-biphenol (**7**) was observed with all the biocatalysts tested. Higher target/buffer integral ratios of the 4,4′-biphenyldisulfate were calculated in the biotransformations containing ASSTB, ASST*Heff*, ASSTC, and ASST*Ddeh*, thus indicating that these enzymes were more prone to transfer a second sulfate group than ASSTA, ASST*Dor*, and ASST*Dfor*. The second sulfate transfer to the smaller hydroxy aromatic molecules (**2**, **3**, **4,** or **6**) was less widespread among the ASSTs. Thus, ASSTB and ASSTC showed exclusively monosulfation with these acceptors. On the other hand, disulfated products were detected when using resorcinol (**3**, ASST*Ddeh* and ASST*Dfor*), hydroquinone (**4**, ASST*Heff*), and 1,3,5-benzenetriol (**6**, ASST*Dfor* and ASST*Dor*). Reactions with catechol only render the monosulfated derivative independently of the enzyme.

Although during the first set of reactions, product formation was monitored until no further increase in absorbance was detected; it was possible that the reactions might have continued, albeit at such slower rate that would not be distinguished during a short period of time. To investigate this possibility, a second batch of biotransformations was set for a longer period of time. From the previously tested group of enzymes, only those with a more restrictive disulfation pattern were selected, i.e., ASSTB and ASSTC, which disulfated only 4,4′-biphenol (**7**), as well as ASST*Ddeh*, which disulfated 4,4′-biphenol (**7**) and resorcinol (**3**). These reactions contained the same acceptor/donor ratio 1:3, although at increased concentrations (10 and 30 mM, respectively). After 16 h of incubation, the biotransformations were quenched and analyzed as before (Fig. S11). As expected, after longer incubation periods, all three enzymes produced new disulfated derivatives. The disulfated counterparts of resorcinol (**3**) and 1,3,5-benzenetriol (**6**) were detected with the three tested ASSTs. In addition, the disulfation products were spotted for hydroquinone (**4**, ASSTB and ASST*Ddeh*), and even for the most demanding one from catechol (**2**, ASST*Ddeh*) (Fig. S12).

#### Activity towards alternative acceptors

**Fig. 7:**
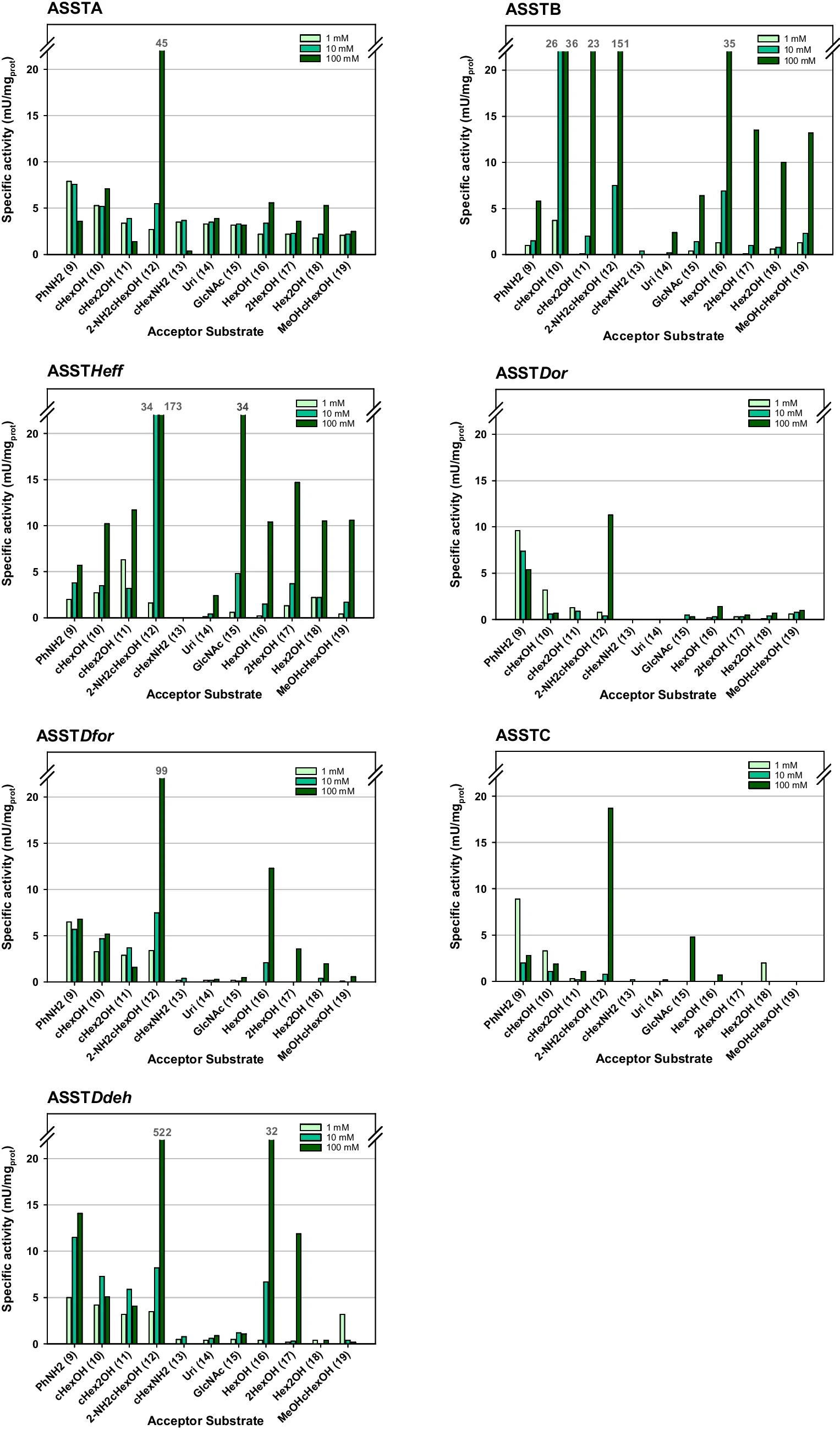
Specific activity towards non-phenolic acceptors (**9**–**19**) displayed by the studied ASSTs. Reaction conditions: Tris/HCl buffer (50 mM, pH 8.0), *p*NPS (1 mM), acceptor substrate (1, 10 or 100 mM), and the purified ASST

As mentioned in the introduction, prior studies demonstrated that certain ASSTs could sulfate aliphatic alcohols, although at a slower rate than aromatic compounds (Funabashi et al. 2010; Hartog and Wever 2015; Islam et al. 2018b; Kaysser et al. 2010; Koryakina et al. 2011; van der Horst et al. 2012). Hence, the substrate scope of the ASSTs was further characterized towards alternative sulfate acceptors **9**–**19** (Fig. 5). This group of compounds included (i) aliphatic alcohols, bearing one or more hydroxy groups (primary and/or secondary), and (ii) amines, both aliphatic and aromatic. Interestingly, sulfate transfer to all these substrates was detected with all ASSTs (Fig. 7). However, the catalytic activities were significantly lower to those determined for the aromatic acceptors, remaining in the mU/mg scale reaching up to 533 mU/mg. Each biocatalyst showed a unique sulfation preference, depending on the substrate and the concentration. Similarly to their behavior towards the first set of substrates, in general, higher concentrations of acceptor in the reaction increased their observed specific activity. Concerning the aliphatic alcohols, the smaller molecules tended to be better accepted for the sulfation reaction. In all cases, the best acceptor substrate was 2-aminocyclohexanol (**12**). When using this substrate, activity values ranged from 522 to 11 mU/mg, for ASST*Ddeh* and ASST*Dor*, respectively. Neither the cyclic or linear structure nor the presence of the hydroxy group in a primary or secondary carbon had an equal effect on the ASSTs substrate selectivity. For instance, the difference between the highest activities obtained towards cyclohexanol (**10**), cyclohexanediol (**11**), 1-hexanol (**16**), *rac-*1,5-hexanediol (**17**), *rac-*2-hexanol (**18**), and *all-rac-*2-methoxycyclohexanol (**19**) was rather small for ASST*Heff* (10–15 mU/mg), slightly bigger for ASSTA and ASSTB (2.5–7.1 mU/mg and 10–36 mU/mg, respectively), and much more significant for ASST*Dor,* ASST*Dfor*, ASSTC, and ASST*Ddeh* (0.5–3.2 mU/mg, 0.6–12.3 mU/mg, no measured activity–3.3 mU/mg, and 0.4–31.5 mU/mg, respectively). When comparing the acceptance of the cyclic vs. linear, only ASST*Dfor* and ASST*Heff* markedly preferred the linear hexanol over the cyclic counterpart. Similarly, most of the ASSTs exhibit comparable activity values for the sulfation of 1-hexanol (**16**) and 2-hexanol (**18**). Nonetheless, ASSTB, ASST*Dfor*, and ASST*Ddeh* favored the primary alcohol over the secondary one (⁓3 × activity). Interestingly, contrary to what was observed with the aromatic alcohols, the presence of a second hydroxy group on the 6-member ring reduced the acceptance of the substrate. All ASSTs, except for ASST*Heff*, showed higher activity towards cyclohexanol (**10**) than towards 1,2-cyclohexanediol (**11**). On the other hand, the most complex alcohol substrates, such as uridine (**14**) and GlcNAc (**15**), showed lower capability to act as acceptors than the smaller molecules. Nonetheless, some exceptions could be distinguished: (i) ASSTA activity towards both aforementioned substrates was comparable to that determined towards the smaller aliphatic alcohols (approx. 3–4 mU/mg), and (ii) GlcNAc was the second-best acceptor substrate for ASST*Heff*, which displayed between two to three times higher activity than towards the other aliphatic alcohols. Regarding the possibility to catalyze the sulfate transfer to amine groups, activity was detected with all the ASSTs when using aniline as acceptor. The formation of *p*NP in these reactions was concentration and enzyme dependent, ranging from 1 to 14 mU/mg with ASSTB (1 mM aniline) and ASST*Ddeh* (100 mM aniline). This clearly indicated the involvement of the enzyme and the substrate on the reaction. Conversely, cyclohexylamine was in general not accepted as substrate and only ASSTA showed evidence to admit it as acceptor substrate (⁓3 mU/mg at 1 mM and 10 mM), which is already interesting considering that the aliphatic amino group is present at the conditions used in its protonated form and therefore not readily available as nucleophile.

## Discussion

In this work, new putative ASSTs were found by performing a pBLAST search using ASSTA as model template. The model protein was chosen due to the synthetic capabilities already described in the literature for this enzyme (Hartog and Wever 2015; Hartog and Wever 2016; van der Horst et al. 2015; van der Horst et al. 2012). Among all the retrieved DNA sequences, five were chosen with decreasing degree of sequence homology: (i) ASST from *Desulfitobacterium dehalogenans* (93% homology); (ii) ASST from *Dehalobacterium formicoaceticum* (86.8% homology); (iii) a new ASST from *Desulfitobacterium hafniense* (71.2% homology); (iv) ASST from *Hungatella effluvii* (58.5% homology); and (v) ASST from *Desulfosporosinos orientis* (58.8% homology), thus increasing the chances to find new enzymatic features and substrate specificities, while ensuring the ASST activity of the novel enzymes. All five enzymes were recombinantly overexpressed in *E. coli*, purified, and characterized. The corresponding results confirmed their capability to perform the sulfate transfer from donor to acceptor aromatic substrates. Concerning their optimal reaction conditions, the highest activity for all of them was measured at pH 8.0 or 9.0. These values were similar to those described for most of the ASST reported in the literature (Hossain et al. 2011; Islam et al. 2018b; Kim et al. 1992; Mozhaev et al. 2002; van der Horst et al. 2012). This might be explained not only by the inherent stability of these enzymes, but also due to a shift of the equilibrium towards the phenolate anion (p*K*a = 9.95) over its protonated counterpart at more alkaline pH, which would facilitate a faster sulfate transfer. Since many potential substrates for ASSTs are poorly water-soluble, organic co-solvents are often required to improve substrate availability in the aqueous phase. Hence, the stability towards co-solvents was established, showing that the best tolerated were 2-propanol, methanol, and DMSO. However, each enzyme retained different percentages of activity in the presence of these solvents. Interestingly, they did not tolerated DMSO nor acetone as well as was published for ASSTA (van der Horst et al. 2012). In general, their behavior in this matter (between 28–58% and 11–27% retained activity, respectively) was closer related to ASSTB (37% and 20%, respectively) (Islam et al. 2018a). In terms of reaction temperature, except of ASST*Heff*, their optimal was between 40 and 45 °C. On the other hand, the recorded melting temperatures of the ASST-homologs, as well as those from ASSTA and ASSTB, were very similar (39–45 °C, at pH 8.0) for all studied proteins. These values were also in accordance with the values previously described in the literature for other ASSTs (Ayuso-Fernández et al. 2014; van der Horst et al. 2012). It is noteworthy that both temperatures, optimal activity and melting, were in the same range (⁓40 °C). Although this might seem counterintuitive, it must be taken into account the stabilizing effect that the presence of substrates in the reaction medium has on the proteins. In the case of ASSTA, the melting temperature of the enzyme was almost 10 °C higher in presence of *p*NPS than in its absence (van der Horst et al. 2012). Thus, this would explain the capability of these enzymes to catalyze best the reaction at temperatures that would denature them in absence of their substrates.

Regarding the study of the kinetic parameters, the sulfate transfer catalyzed by all ASSTs followed a Michaelis–Menten behavior for the donor substrate, hence exhibiting a linear dependency of the reaction rate on *p*NPS concentration. Under our reaction conditions, the catalytic efficiency value calculated for ASSTA (*p*NPS/PhOH) was higher than those previously described (Brodsky et al. 2022), albeit on the same order of magnitude (83.9 and 42.2 mM^−1^ s^−1^, respectively). However, when using catechol as acceptor, the catalytic efficiency value obtained in this study was almost one order the magnitude higher than the one previously reported (215.8 and 24.9 mM^−1^ s^−1^, respectively). Nevertheless, it must be kept in mind that direct comparison of the obtained data with that previously described in the literature is not feasible, since not exactly the same assay conditions were used. Likewise, as for ASSTA, the acceptor substrate (either phenol or catechol) present also influenced the *V*_max_ and *K*_M_ of the investigated ASSTA-homologs, hence influencing their turnover number and catalytic efficiency. This difference was particularly pronounced in case of ASST*Dor*, with which the catalytic efficiency towards *p*NPS with catechol was approximately 40-fold higher than when using phenol as acceptor substrate. This could be explained due to the marked selectivity of this enzyme towards catechol. Comparison of their catalytic efficiency reveals a difference of up to two orders of magnitude. ASST*Ddeh* displayed the highest *k*_cat_/*K*_M_ (mM^−1^ s^−1^) when transferring the sulfate group to catechol, being between twofold and 53-fold more efficient than the other ASSTs studied. In the case of phenol sulfation, ASSTA and ASST*Ddeh* showed the highest efficiency (83.9 and 63.2 mM^−1^ s^−1^, respectively), while ASST*Dor* had the lowest *k*_cat_/*K*_M_ (0.6 mM^−1^ s^−1^).

A variety of phenolic compounds were tested as potential sulfate acceptor for these enzymes. The results were in line with those found for known ASSTs (Ayuso-Fernández et al. 2014; Kolaříková et al. 2022; Mozhaev et al. 2002; van der Horst et al. 2015; van der Horst et al. 2012), thus demonstrating their capability to sulfate these molecules. Although most ASSTA-homologs showed a broad acceptance scope, their substrate preference was specific to each enzyme. For instance, ASST*Dor* preferred clearly catechol (**2**), while such a strong preference was not detected for any other enzyme, being ASSTB the least selective in terms of aromatic acceptors. Nonetheless, in general, activities towards catechol (**2**) and 1,2,4-bezenetriol (**5**) were the highest for all ASSTs. This was in accordance with the studies performed with dihydroxyphenolic acids by Valentová and coworkers (Kolaříková et al. 2022), who hypothesized that the two adjacent hydroxyl groups could influence the activity by the binding of the enzyme to one of them, while realizing the sulfate transfer to the alcohol in *ortho-*position. Acceptance of di- or trihydroxyphenols with a second alcohol in *meta*- or *para*-position was similar in range, although considerably less preferred than the best accepted 1,2-dihydroxyphenyl substrate. Bulkier molecules with more than one phenyl ring were also accepted. This can be very interesting from a synthetic point of view, since it opens the possibility to apply these ASSTs for the regioselective synthesis of relevant sulfated aromatic metabolites (Marhol et al. 2013; Purchartová et al. 2013, 2015; Valentová et al. 2017; van der Horst et al. 2015). Transfer of more than one sulfate group to the same molecule was verified. All the analyzed di- and trihydroxy aromatic substrates proved to be able to undergo disulfation. Contrarily, no trisulfated compounds were detected. Similarly to the activity patterns, the selectivity towards disulfation was characteristic of each enzyme. For instance, ASST*Dor*, ASST*Dfor*, and ASSTC preferred the disulfation of compounds bearing a 1,3- over 1,4-hydroxyl group pattern, while ASST*Heff*, ASST*Ddeh*, and ASSTB had the opposite preference. However, some common trends were observed: the only substrate onto which all the enzymes could transfer two sulfate groups was 4,4′-biphenol (**7**). This is quite likely due to the distance between both alcohol groups and the symmetry of the molecule. On the other hand, the disulfated product of catechol was not detected in any of the reactions performed during short periods of time (below 3 h). A reasonable explanation would be the difficulty of adding a second sulfate group to a position so close to where a sterically demanding prior sulfate group was transferred. Unexpectedly, the corresponding *m/z* ratio for this disulfated compound (268 [*M*-H]^−^, 134 [*M*-2H]^2−^) was detected when analyzing reactions catalyzed by ASSTB after 16 h of incubation. Since only the monosulfated catechol, and not the substrate, was detected after 3 h, this indicates the slow rate for the second sulfate transfer, which requires significantly longer periods of time. To the best of our knowledge, the possibility to disulfate polyhydroxyphenols was only demonstrated with ASSTs from *D. hafniense* (Marhol et al. 2013; van der Horst et al. 2015) and *H. ochraceum* (Ayuso-Fernández et al. 2014). However, considering the results obtained here, this could be a common characteristic among ASSTs, or at least among those closely related to ASSTA.

The biological role of sulfated carbohydrates, steroids, and small molecules makes them very desirable due to their potential application in therapeutics (Mueller et al. 2015; Wieboldt and Läubli 2022; Zeng et al. 2019). Hence, despite the clear predilection of ASSTs towards aromatic substrates, efforts to apply them for the obtaining of aliphatic alcohols were attempted. The successful application of ASSTs for this task is particularly appealing due to their capacity to use cheap sulfate donors (*p*NPS, *p*MUS, etc.), rather than expensive and labile ones, such as PAPS. In this sense, a few ASSTs have demonstrated their ability to catalyze the sulfate transfer onto aliphatic molecules. For instance, *Streptomyces* sp. ASSTs, LipB and Cpz4, were proved to catalyze the sulfation of the sugar moiety of nucleoside derivatives (Funabashi et al. 2010; Kaysser et al. 2010). Other examples include the ASSTs from *D.* *hafniense*, ASSTA and ASSTB. Prior studies demonstrated the sulfation of hydroxyl groups in an aliphatic position of small molecules by ASSTA (Hartog and Wever 2015; van der Horst et al. 2012) and the acceptance of sugars by ASSTB (Islam et al. 2018a, 2018b). As aforementioned, one of the main driving factors to choose ASSTA as homology model template was the chance to discover enzymes with similar or improved behavior towards these acceptor substrates. The specific activity results showed that, although much slower than with phenolic substrates, all the ASSTs studied catalyzed the sulfate transfer towards aliphatic alcohols. Particularly good results were obtained with the 2-aminocyclohenanol (522 to 11 mU/mg). A plausible explanation could be the easier deprotonation of the alcohol facilitated by the adjacent amine, thus favoring the transfer of the sulfate group. Additionally, it is noteworthy that ASST*Heff* showed approximately 6 times higher activity towards GlcNAc than ASSTB. This makes this enzyme an interesting protein engineering candidate for the obtaining of sulfated sugars and oligosaccharides. Finally, all the enzymes displayed activity towards one or both of the amine compounds, i.e., aniline and cyclohexane amine, being the aromatic substrate preferred over the aliphatic one. As far as we are aware, this is the first study where ASST activity was detected using compounds bearing only amine groups (without any hydroxyl group) as acceptor substrate, indicating the ability of the ASSTs to sulfate not only alcohols, but also amines.

In conclusion, in this work, five new ASSTs were obtained, characterized, and their synthetic potential confirmed. Hence, this panel of investigated enzymes allows for the selection of the best candidate, depending on the sulfate transfer needs, i.e., specific activity, catalytic efficiency, selectivity towards aromatic acceptors, transfer of several sulfate groups, sulfation of aliphatic alcohols, and/or amines.

## Supplementary Information

Below is the link to the electronic supplementary material.

Supplementary file1 (PDF 2429 KB)

## Data Availability

Correspondence and material requests should be addressed to Prof. Wolfgang Kroutil (wolfgang.kroutil@uni-graz.at).
