# Supplementary material for: Biocatalytic sulfation of aromatic and aliphatic alcohols catalyzed by arylsulfate sulfotransferases
Source: Appl Microbiol Biotechnol. 2024 Nov 19;108:520. doi: 10.1007/s00253-024-13354-5 (PMC11576661; doi:10.1007/s00253-024-13354-5)
Supplement: Supplementary file 1 [file 253_2024_13354_MOESM1_ESM.pdf]

Supporting Information

Biocatalytic sulfation of aromatic and aliphatic alcohols catalyzed by arylsulfate sulfotransferases

Isabel Oroz-Guinea,[1,2,3] Marko Rath,[1,2] Isabelle Tischler,[2] Klaus Ditrich,[4] Michael Breuer,[4] Doreen Schachtschabel,[4] Wolfgang Kroutil [1,2,3,5]

[1] Austrian Centre of Industrial Biotechnology c/o University of Graz Heinrichstrasse 28, 8010 Graz (Austria).

[2] Institute of Chemistry University of Graz, NAWI Graz, Heinrichstrasse 28, 8010 Graz (Austria).

[3] Field of Excellence BioHealth University of Graz Heinrichstrasse 28, 8010 Graz (Austria).

[4] BASF SE White Biotechnology Research, Carl-Bosch-Strasse 38, 67056 Ludwigshafen (Germany).

[5] BioTechMed Graz Heinrichstrasse 28, 8010 Graz (Austria).

Corresponding author: Wolfgang Kroutil, wolfgang.kroutil@uni-graz.at

Table S1 Summary of the sequences retrieved using BLASTp with ASSTA as protein template

ASST / Source μorganism	Sequence identity	Amino acids	NCBI
ASST *Desulfitobacterium dehalogenans* ATCC 51507*	93.2	629	AFM01510.1
ASST *Desulfitobacterium dichloroeliminans*	87.6	628	WP_015263010.1
ASST *Dehalobacterium formicoaceticum**	86.8	628	WP_089608557.1
ASST *Desulfosporosinus lacus*	85.0	627	WP_073033341
ASST *Desulfosporosinus* sp. HMP52	83.7	628	WP_034601251.1
ASST *Desulfosporosinus meridiei*	84.6	628	WP_014902777.1
ASST *Desulfosporosinus hippei*	83.1	628	WP_092334873.1
ASST *Desulfosporosinus acididurans*	77.3	628	WP_047811757.1
ASST *Desulfosporosinus* sp. FKA	76.5	629	WP_088186734.1
ASST *Desulfitobacterium dehalogenans*	76.1	629	WP_014793958.1
ASST *Desulfitobacterium dichloroeliminans*	75.6	629	WP_015260940.1
ASST *Desulfitobacterium hafniense**	71.2	626	KTE91359.1
ASST *Desulfitobacterium chlororespirans*	71	626	WP_072774114.1
ASST *Clostridiales bacterium*	70	636	PWM49771.1
ASST *Bittarella massiliensis*	64.9	625	WP_059003491.1
Clostridium hypothetical protein	64.7	625	WP_021658034.1
ASST *Desulfohalotomaculum alkaliphilum*	60.4	622	WP_114638903.1
ASST *Lachnoclostridium* sp. An14	57.2	628	WP_087224291.1
ASST *Niameybacter massiliensis*	58.7	619	WP_053984844.1
ASST *Desulfohalotomaculum alkaliphilum*	58.4	618	WP_031517900.1
ASST *Desulfosporosinus orientis**	58.8	621	WP_014184280.1
ASST *Desulfotomaculum ferrireducens*	58.1	618	WP_077713083.1
ASST *Hungatella hathewayi*	59	624	WP_055659810.1
ASST *Desulfosporosinus orientis**	58.3	621	WP_014187168.1
ASST *Niameybacter massiliensis*	56.6	626	WP_053984585.1
ASST *Desulfitobacterium dichloroeliminans*	58.9	623	WP_015263770.1
ASST *Desulfotomaculum ferrireducens*	57	621	WP_077715208.1
ASST *Hungatella effluvia**	58.5	624	WP_110321498.1
ASST *Niameybacter massiliensis*	55.2	622	WP_053983873.1

* Selected DNA sequences on this work

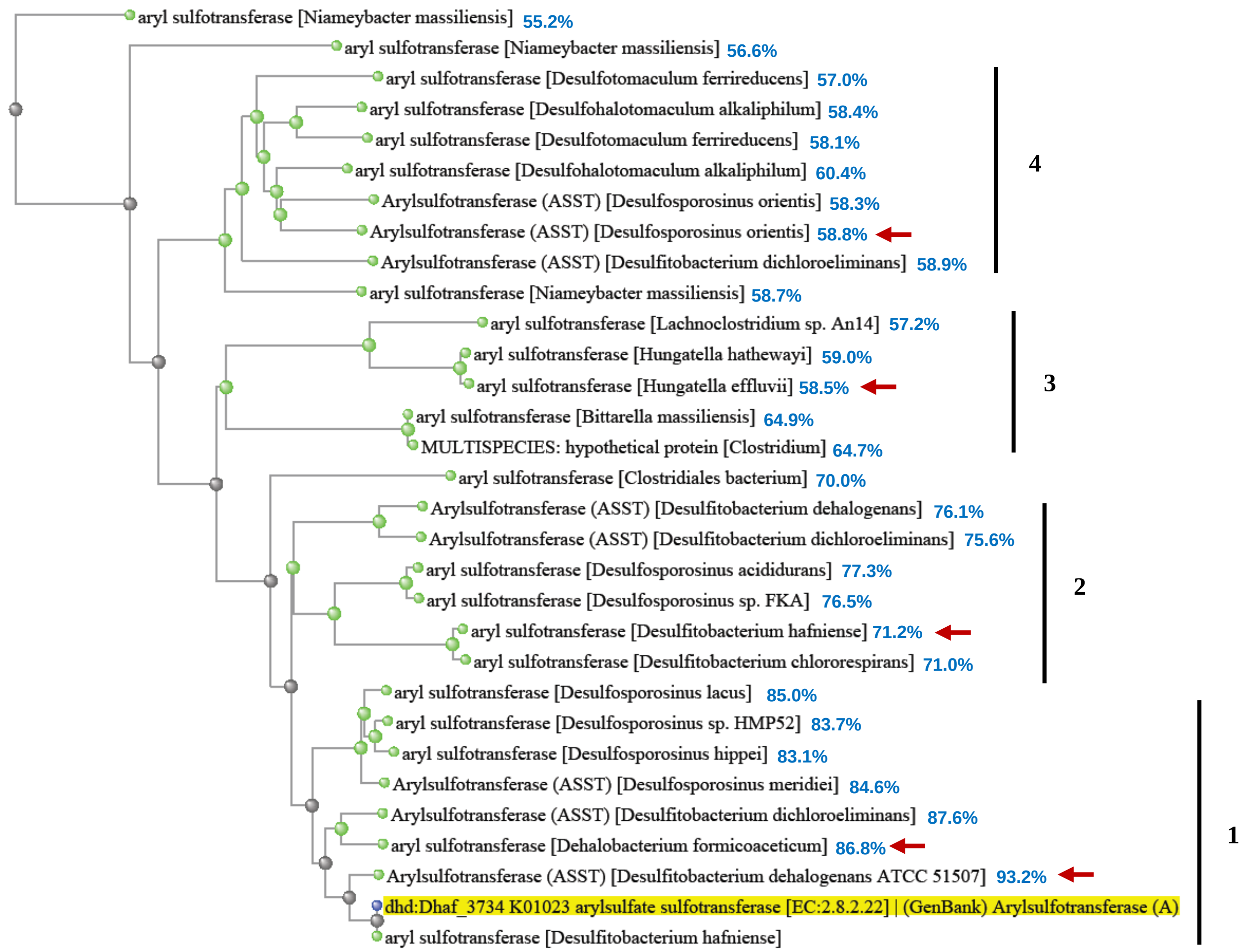

Fig. S1 Homology tree showing the sequence similarity displayed by the proteins retrieved from the BLAST search using ASSTA from *D. hafniense* as protein template. The four main branches that constitute the tree are numbered to facilitate their differentiation. The model protein is highlighted in yellow and the homologous proteins chosen are marked with red arrows. Homology percentages with ASSTA are written in blue next to all the protein names

Internal

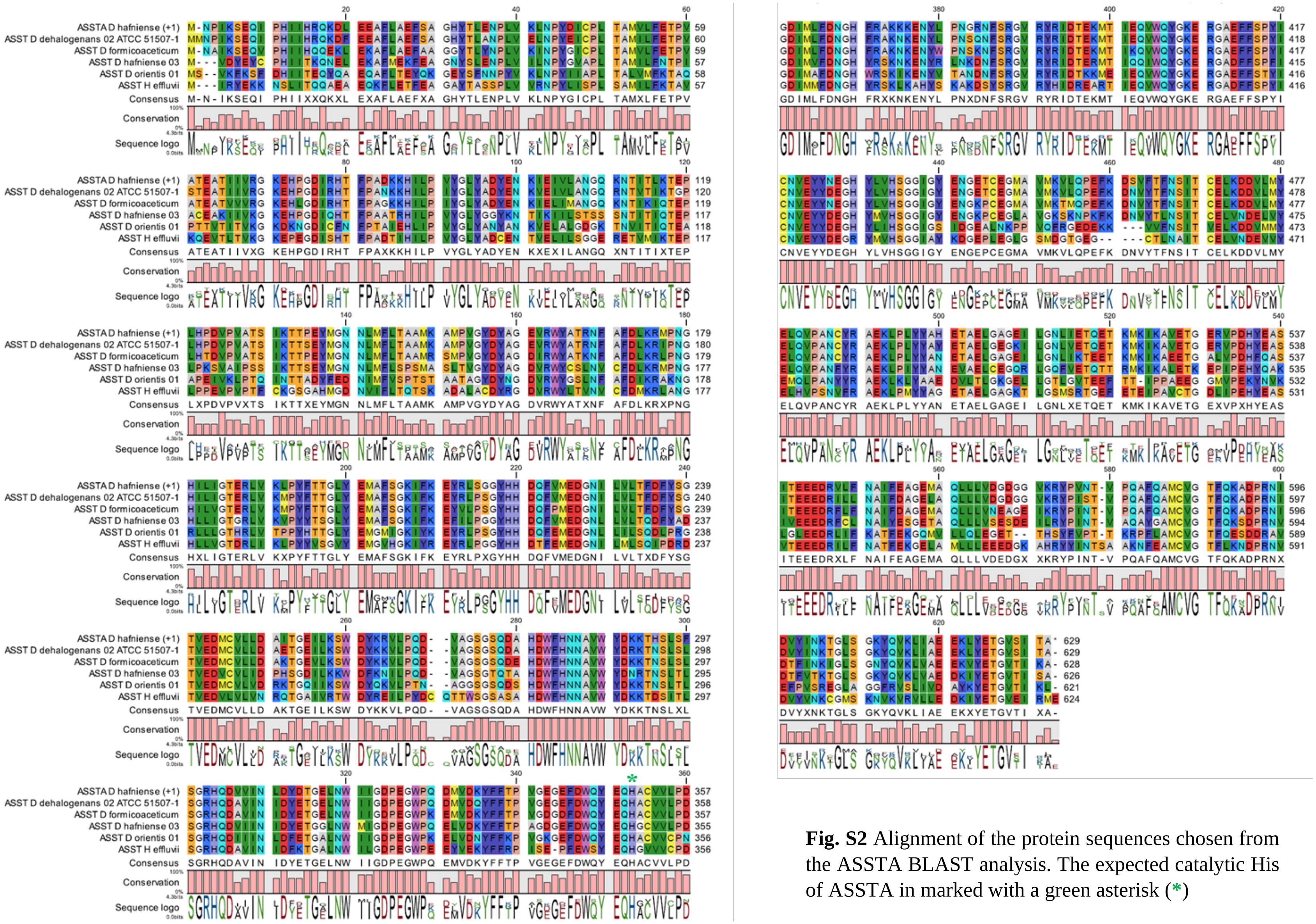

Fig. S2 Alignment of the protein sequences chosen from the ASSTA BLAST analysis. The expected catalytic His of ASSTA in marked with a green asterisk (*)

Table S2 Summary of the strains and culture conditions utilized for the overexpression of the ASSTs

Enzyme	ASSTA	ASSTB	ASST*Heff*	ASST*Dor*	ASST*Dfor*	ASSTC	ASST*Ddeh*
**pEG**[a]	pEG548	pEG549	pEG559	pEG558	pEG556	pEG557	pEG555
**Vector**	pET26b(+)	pET28a(+)	pET28a(+)	pET28a(+)	pET28a(+)	pET28a(+)	pET28a(+)
**Restriction sites**	*Sac*I and *Sal*I	*Nco*I and *Sal*I	*Nco*I and *Sal*I	*Nco*I and *Sal*I	*Nco*I and *Sal*I	*Nco*I and *Sal*I	*Nco*I and *Sal*I
**Tag**	C-term His-tag	C-term His-tag	C-term His-tag	C-term His-tag	C-term His-tag	C-term His-tag	C-term His-tag
**Expression strain**	BL21(DE3)	BL21(DE3)	BL21(DE3)	BL21(DE3)	BL21(DE3)	BL21(DE3)	BL21(DE3)
**Media**	LB	LB	LB	LB	LB	LB	LB
**$[\text{Antibiotic}]_{cult}$**	Kan (50 µg/ml)	Kan (50 µg/ml)	Kan (50 µg/ml)	Kan (50 µg/ml)	Kan (50 µg/ml)	Kan (50 µg/ml)	Kan (50 µg/ml)
**O/Nc volume, temperature and shaking**	10 ml, 37 °C, 120 rpm	10 ml, 37 °C, 120 rpm	10 ml, 37 °C, 120 rpm	10 ml, 37 °C, 120 rpm	10 ml, 37 °C, 120 rpm	10 ml, 37 °C, 120 rpm	10 ml, 37 °C, 120 rpm
**Usual culture volume**	500 ml	500 ml	500 ml	500 ml	500 ml	500 ml	500 ml
**Inoculation volume**	1 % (5 ml)	1 % (5 ml)	1 % (5 ml)	1 % (5 ml)	1 % (5 ml)	1 % (5 ml)	1 % (5 ml)
**Culture temperature and shaking**	37 ºC, 140 rpm	37 ºC, 140 rpm	37 ºC, 140 rpm	37 ºC, 140 rpm	37 ºC, 140 rpm	37 ºC, 140 rpm	37 ºC, 140 rpm
**Induction OD_{600}**	~0.6	~0.6	~0.6	~0.6	~0.6	~0.6	~0.6
**Inducer**	IPTG (1 mM)	IPTG (0.4 mM)	IPTG (1 mM)	IPTG (1 mM)	IPTG (1 mM)	IPTG (1 mM)	IPTG (1 mM)
**Induction time, temperature and shaking.**	O/N, 20 ºC, 120 rpm	O/N, 30 ºC, 140 rpm	O/N, 20 ºC, 120 rpm	O/N, 20 ºC, 120 rpm	O/N, 20 ºC, 120 rpm	O/N, 20 ºC, 120 rpm	O/N, 20 ºC, 120 rpm
**Purification**	Ni^{2+}-NTA	Ni^{2+}-NTA	Ni^{2+}-NTA	Ni^{2+}-NTA	Ni^{2+}-NTA	Ni^{2+}-NTA	Ni^{2+}-NTA

[a] pEG refers to the internal plasmid numbering: pEG stand for plasmid of the Elk Group.

Table S3 Expected molecular weights and theoretical molar extinction coefficients for the different overexpressed ASSTs

ASST	Molecular weight (kDa)	Theoretical molar extinction coefficient [M^{-1} cm^{-1}]
ASSTA	76.0	93360
ASSTB	74.0	110310
ASST*Heff*	70.7	99290
ASST*Dor*	70.5	97650
ASST*Dfor*	71.4	97770
ASSTC	71.0	96160
ASST*Ddeh*	71.6	94640

Table S4 Determined *p*NP molar extinction coefficient under the different reaction conditions employed

Buffer	pH	Wavelength (nm)	Co-solvent (10%, v/v)	Molar extinction coefficient (ε) mM^{-1} cm^{-1}
Bis Tris	5.5	350 nm	—	5.0
	6.0		—	5.0
	7.0	410 nm	—	8.4
Na^+ phosphate	7.0	410 nm	—	11.1
	8.0		—	15.7
Tris / HCl	8.0	410 nm	—	15.6
	9.0		—	16.7
Na^+ carbonate	9.0	410 nm	—	16.9
	10.0		—	16.9
	10.5		—	16.8
AMP	9.0	410 nm	—	17.4
	10		—	17.4
	10.5		—	17.7
Tris / HCl	8.0	410	DMSO	18.7
			Acetone	18.3
			Methanol	17.8
			2-Propanol	19.0
			DMF	19.2
			1,2-$(MeO)_2$Et	18.8

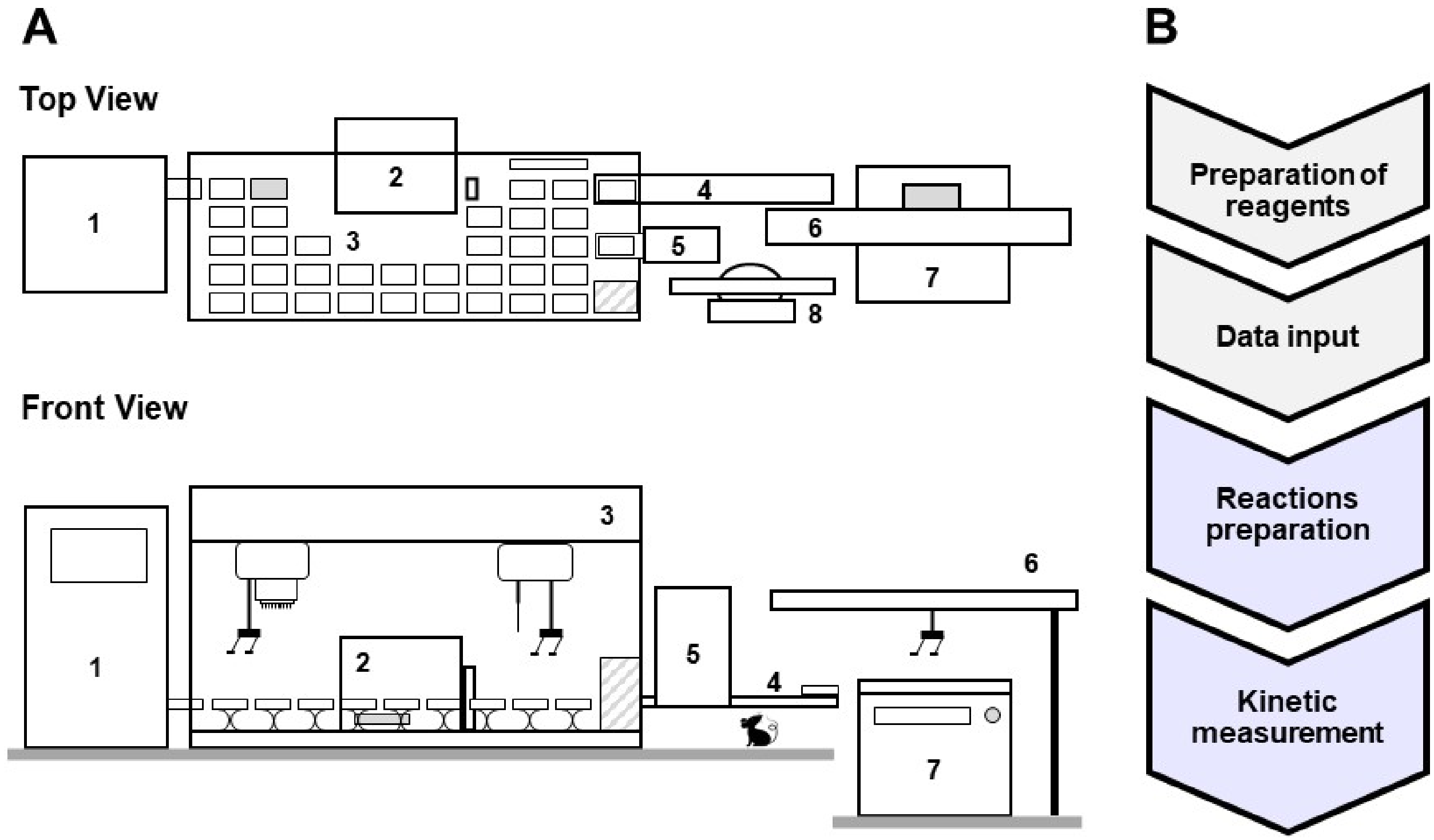

Fig. S3 (**A**) Schematic representation of the high throughput robotic platform (Xavier), including (**1**) an SBS-format plates incubator (Cytomat 2C450), (**2**) an UV/vis and fluorescence microtiter plate (MTP) reader (FLUOstar Omega, BMG), (**3**) a liquid handling robot (Biomek i7 Hybrid, Beckman Coulter), (**4**) MTP shuttle (Servo Shuttle, Beckman Coulter), (**5**) colony detection module (Visual ALP, Amplius), (**6**) BRT II and (**7**) refrigerated centrifuge (Rotanta 460, Hettich). These different devices are controlled and integrated through (**8**) Biomek and DART 2.0 software (representation of the PC on the front view was avoided for clarity). (**B**) Workflow for measuring the ASSTs Michaelis-Menten kinetics (grey background: steps performed manually; blue background: automated steps performed by the robotic platform)

Table S5 FIA-MS method applied for the detection of mono-, di- and trisulfated compounds synthesized by ASSTs

**Column:**	no column, SecurityGuard™ C18 cartridge
**Column temperature:**	not controlled
**Eluent composition:**	H_2O/MeOH 50:50, isocratic
**Eluent flow rate:**	0.5 mL/min
**Run time:**	1.4 min
**Injection volume:**	5 µl
**Ionization mode:**	API-ES, negative polarity
**Drying gas temperature:**	300 ºC
**Drying gas flow:**	6.0 L/min
**Nebulizer pressure:**	15 psig (2.0 bar)
**Capillary voltage:**	3000 V
**Signal 1 (scan):**	100% cycle time
	Mass range 30-500
	Fragmentor 70 V

Note: All samples were analyzed in scan mode and the chromatograms of the target masses were extracted in post-processing

Internal

Table S6 Target masses and internal standards used for the detection of the sulfated compounds synthesized in the biotransformations with substrates 2-7

**Analyte**	**Mass (*m/z*)**
PIPES	301 [*M*-H]$^-$
*p*NPS	218 [*M*-H]$^-$
*p*NP	138 [*M*-H]$^-$
Cathechol (**2**)	109 [*M*-H]$^-$
Monosulfated **2**	189 [*M*-H]$^-$
Disulfated **2**	269 [*M*-H]$^-$, 134 [*M*-2H]$^{2-}$
Resorcinol (**3**)	109 [*M*-H]$^-$
Monosulfated **3**	189 [*M*-H]$^-$
Disulfated **3**	269 [*M*-H]$^-$, 134 [*M*-2H]$^{2-}$
Hydroquinone (**4**)	109 [*M*-H]$^-$
Monosulfated **4**	189 [*M*-H]$^-$
Disulfated **4**	269 [*M*-H]$^-$, 134 [*M*-2H]$^{2-}$
1,2,4-benzenetriol (**5**),	125 [*M*-H]$^-$
Monosulfated **5**	205 [*M*-H]$^-$
Disulfated **5**	285 [*M*-H]$^-$, 142 [*M*-2H]$^{2-}$
Trisulfated **5**	365 [*M*-H]$^-$
1,3,5-benzenetriol (**6**)	125 [*M*-H]$^-$
Monosulfated **6**	205 [*M*-H]$^-$
Disulfated **6**	285 [*M*-H]$^-$, 142 [*M*-2H]$^{2-}$
Trisulfated **6**	365 [*M*-H]$^-$
4,4′-dihydroxybiphenyl (**7**)	185 [*M*-H]$^-$
Monosulfated **7**	265 [*M*-H]$^-$
Disulfated **7**	345 [*M*-H]$^-$, 172 [*M*-2H]$^{2-}$

Internal

Table S7 HPLC-MS method applied for the detection of mono-, di- and trisulfated compounds synthesized by ASSTs

**Column:**	*Phenomenex* Luna C18 column, SecurityGuard™ C18 cartridge
**Column temperature:**	not controlled
**Eluent composition:**	H_2O/MeOH 50:50, isocratic
**Eluent flow rate:**	1.0 mL/min
**Run time:**	15 min
**Injection volume:**	5 µl
**Ionization mode:**	API-ES, negative polarity
**Drying gas temperature:**	350 ºC
**Drying gas flow:**	10.0 L/min
**Nebulizer pressure:**	35 psig (2.4 bar)
**Capillary voltage:**	3000 V
**Signal 1 (scan):**	100% cycle time
	Mass range 35-500
	Fragmentor 40 V

Note: All samples were analysed in scan mode and the chromatograms of the target masses were extracted in post-processing

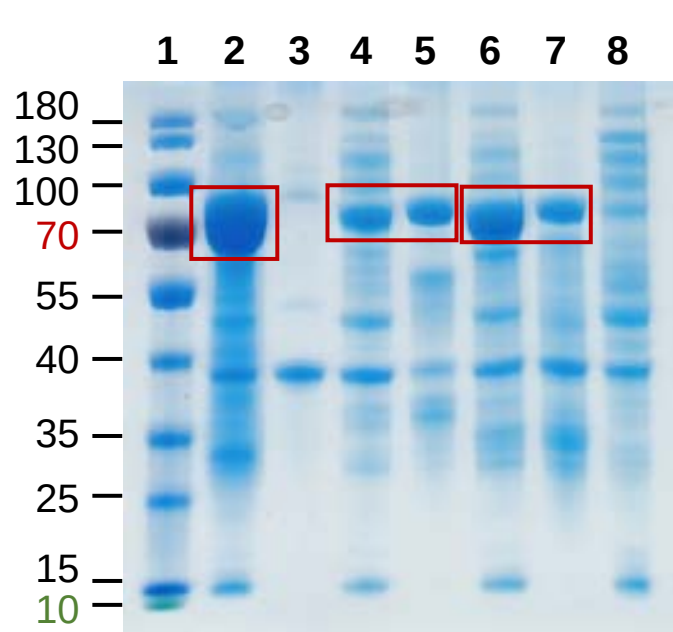

1. Molecular weight ladder
2. CFE ASST*Heff*
3. IB ASST*Heff*
4. CFE ASST*Dor*
5. IB ASST*Dor*
6. CFE ASST*Dfor*
7. IB ASST*Dfor*
8. CFE (-)

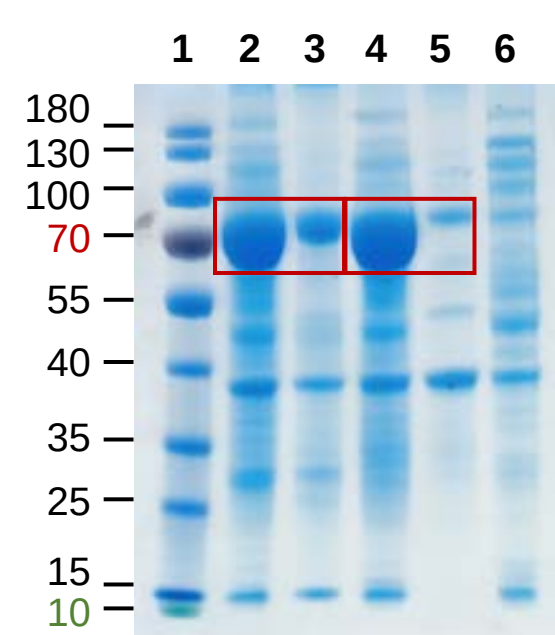

1. Molecular weight ladder
2. CFE ASSTC
3. IB ASSTC
4. CFE ASST*Ddeh*
5. IB ASST*Ddeh*
6. CFE (-)

Fig. S4 Overexpression of the ASSTs displaying sequence homology with ASSTA. **CFE**: cell free extract; **IB**: inclusion bodies; **(-)**: negative control. The expected molecular weights are described in **Table S3**

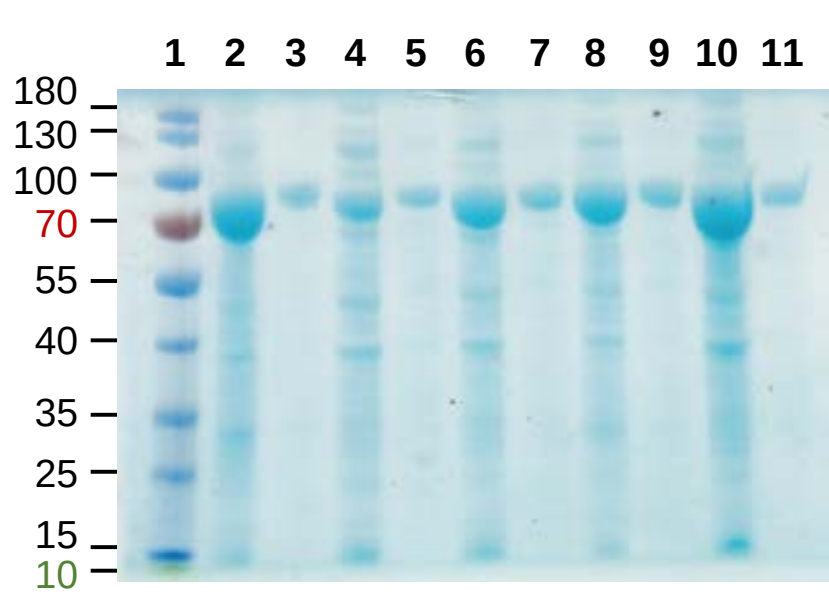

1. Molecular weight ladder
2. CFE ASST*Heff*
3. Purified ASST*Heff*
4. CFE ASST*Dor*
5. Purified ASST*Dor*
6. CFE ASST*Dfor*
7. Purified ASST*Dfor*
8. CFE ASSTC
9. Purified ASSTC
10. CFE ASST*Ddeh*
11. Purified ASST*Ddeh*

Fig. S5 IMAC purification of the ASSTs displaying sequence homology with ASSTA. **CFE**: cell free extract; **(-)**: negative control. The expected molecular weights are described in **Table S3**

Table S8 Activity measured for the different ASSTs

ASST	Activity U/ml$_{CFE}$ Substrate 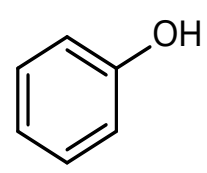 **Phenol**	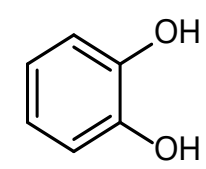 **Catechol**	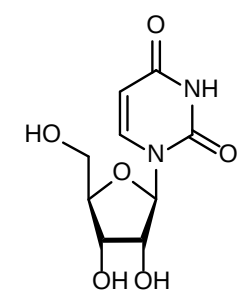 **Uridine**	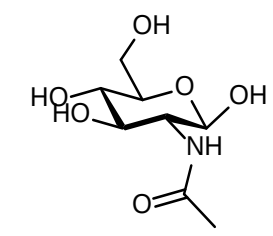 **GlcNAc**
ASST*Heff*	1.7	20.0	n.d.	n.d.
ASST*Dor*	0.1	2.0	n.d.	n.d.
ASST*Dfor*	7.4	26.0	n.d.	n.d.
ASSTC	0.2	1.5	n.d.	n.d.
ASST*Ddeh*	22.3	135.9	n.d.	n.d.
ASSTA	0.7	3.4	n.d.	n.d.
ASSTB	11.6	16.6	n.d.	n.d.

n.d: no activity detected

Reactions conditions: Tris/HCl buffer (50 mM, pH 8.0), *p*NPS (1 mM), acceptor substrate (1 mM) and the ASST-containing CFE. Reaction progress was followed spectrophotometrically at 410 nm

Table S9 Melting temperature values measured by thermofluor assay. Each sample contained Sypro® Orange as fluorescent dye (2.5 µl in 50 mM MES buffer pH 7.0) and the corresponding ASST (1 mg/ml final conc.) dissolved in buffer (Tris/HCl 50 mM at pH 8.0 or 9.0, 25 µl final volume)

	T_m (°C) pH 8.0	pH 9.0
**ASSTA**	44.5 ± 0.7	40.0 ± 0.0
**ASSTB**	43.0 ± 0.0	38.3 ± 0.6
**ASSTC**	39.3 ± 0.6	35.7 ± 0.6
**ASST*Heff***	38.7 ± 0.6	35.7 ± 2.1
**ASST*Dfor***	40.7 ± 0.6	38.0 ± 0.0
**ASST*Dor***	38.0 ± 1.0	38.0 ± 0.0
**ASST*Ddeh***	39.0 ± 0.0	36.0 ± 0.0

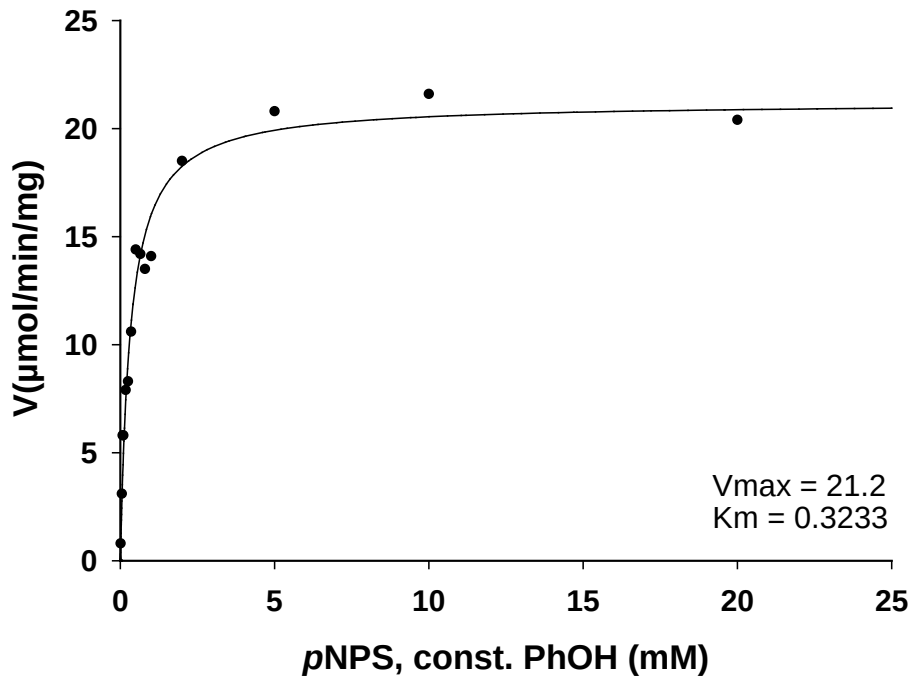

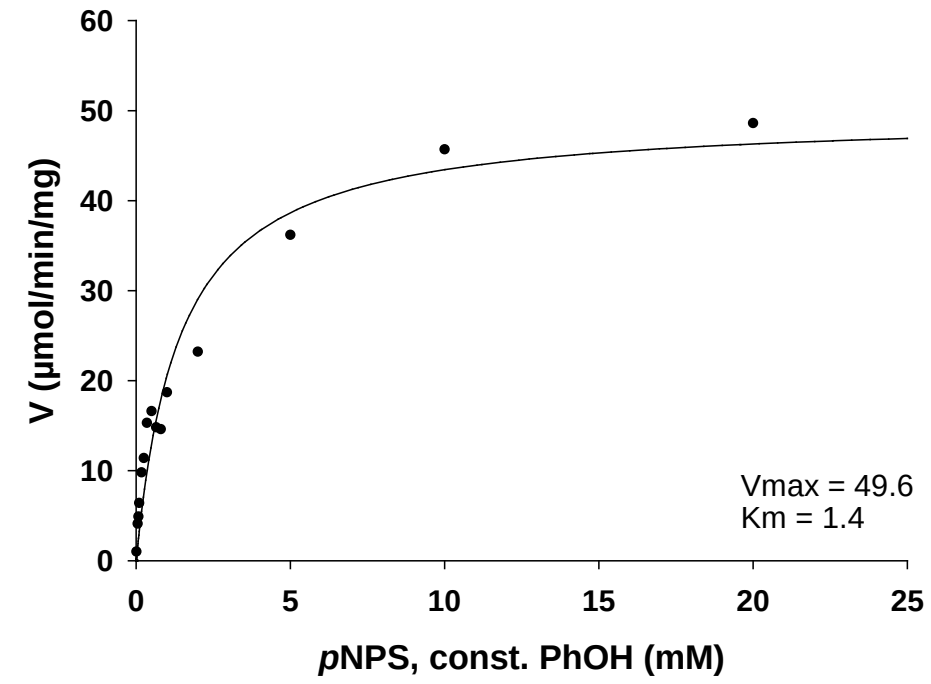

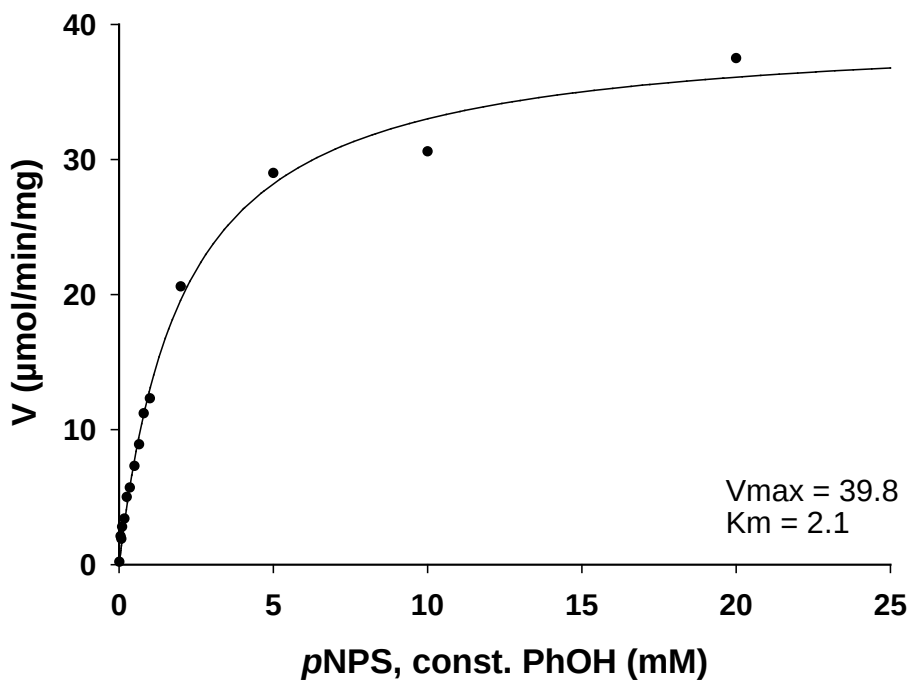

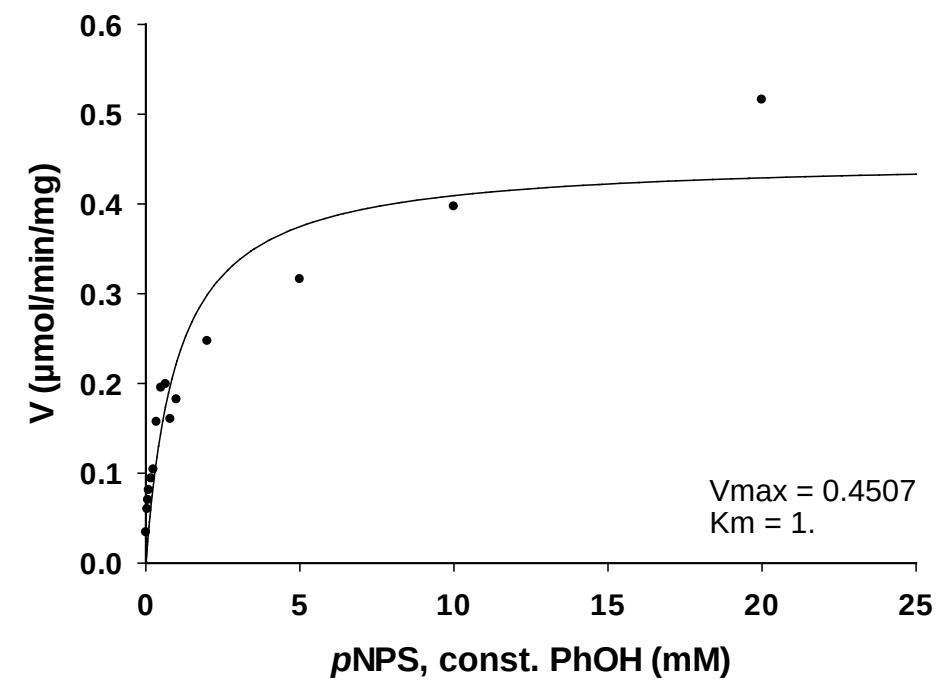

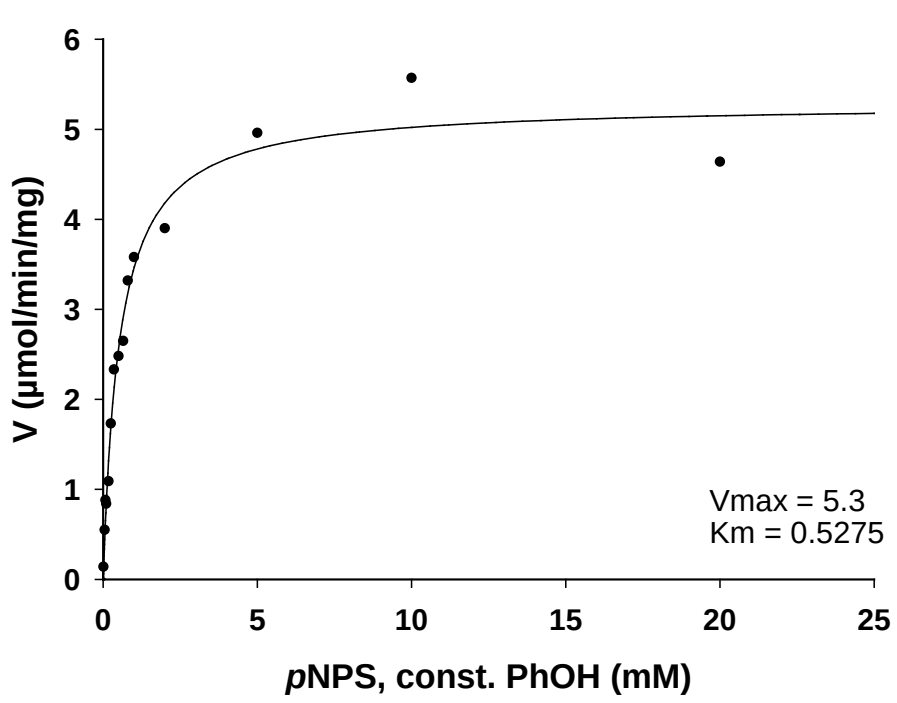

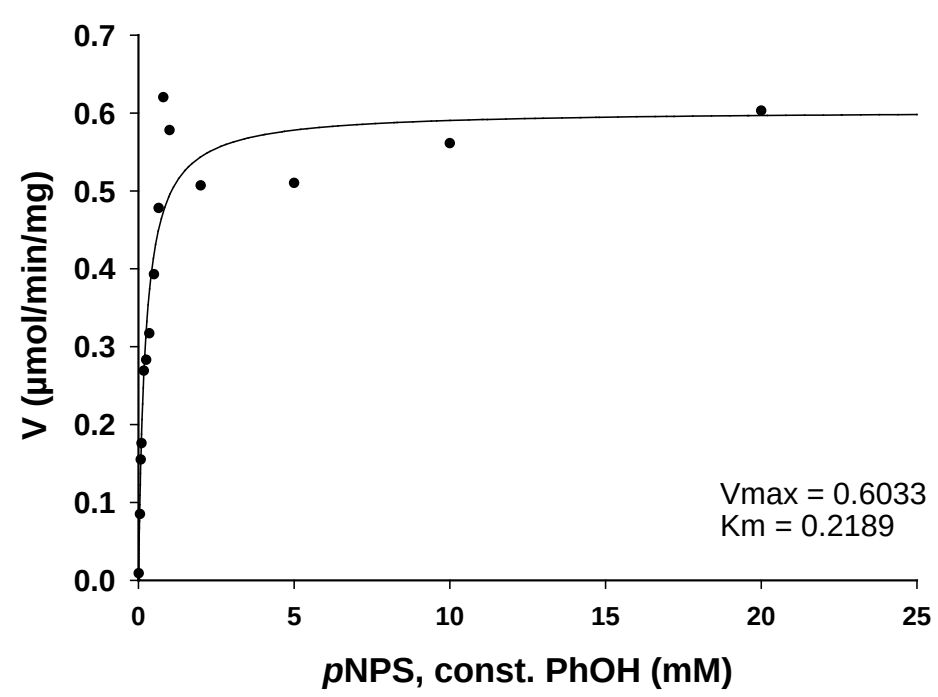

ASST*Ddeh*

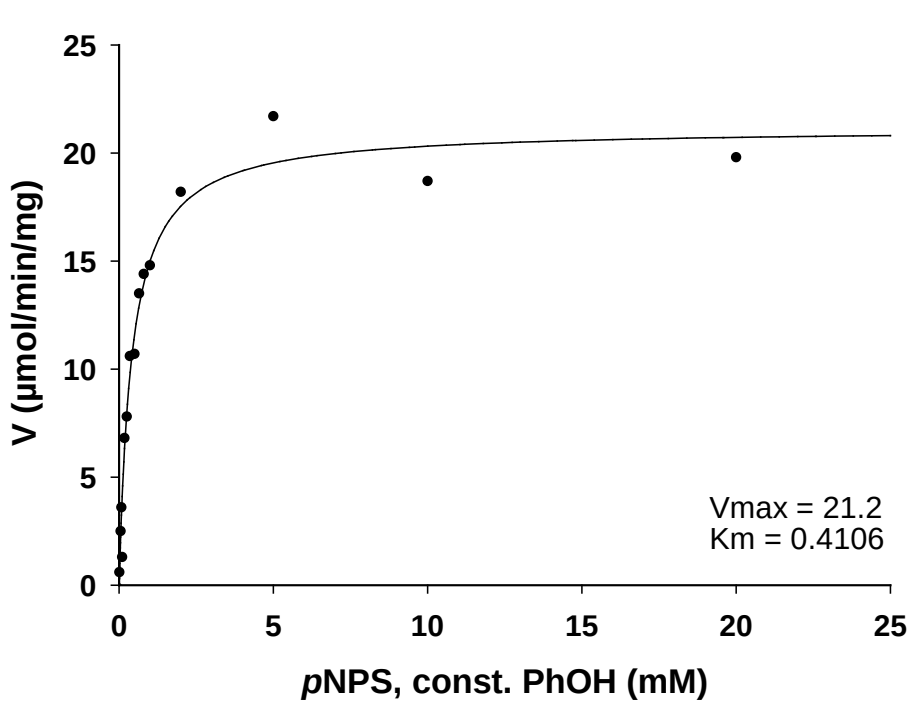

Fig. S6 Apparent kinetic parameters of ASSTA, ASSTB, ASST*Heff*, ASST*Dor*, ASST*Dfor*, ASSTC, and ASST*Ddeh* for *p*NPS. Reaction conditions: Tris/HCl buffer (50 mM, pH 8.0), *p*NPS (0-20 mM), phenol (**1**) (100 mM) and the purified ASST (50 nM)

Internal

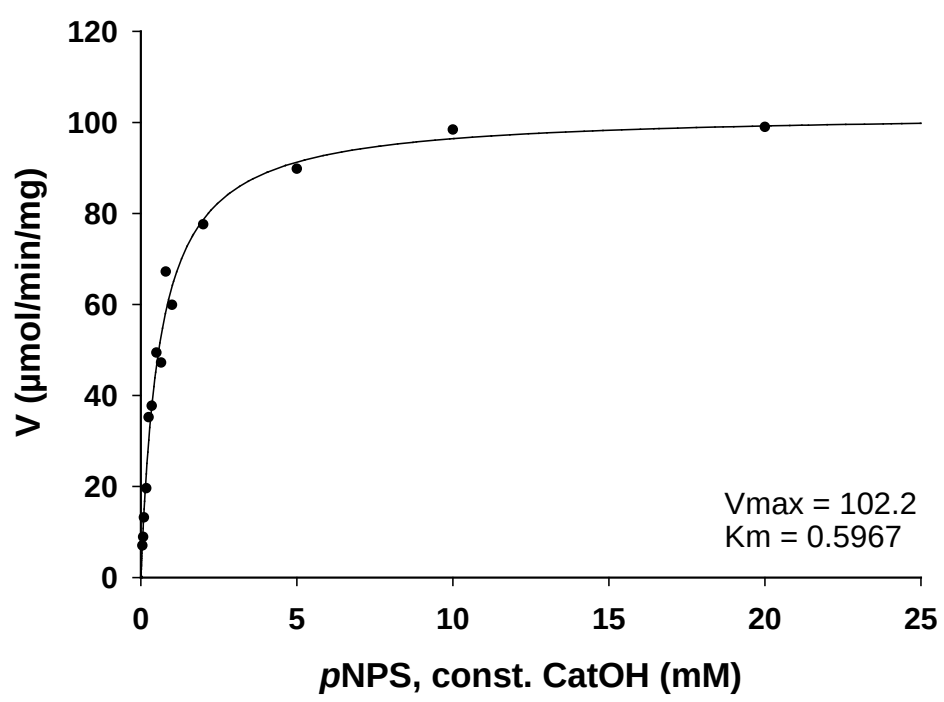

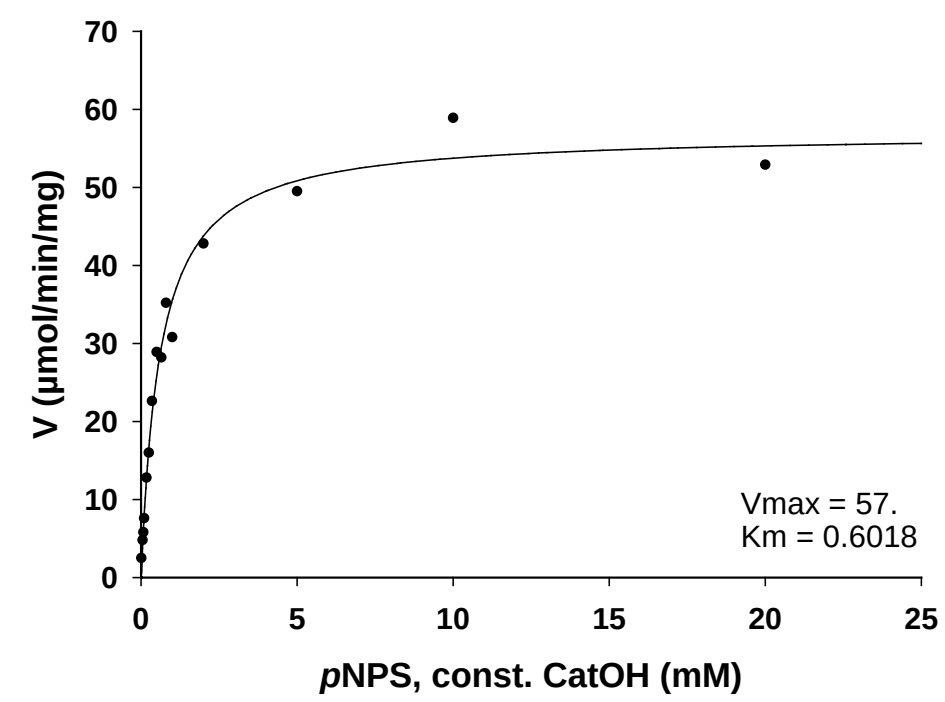

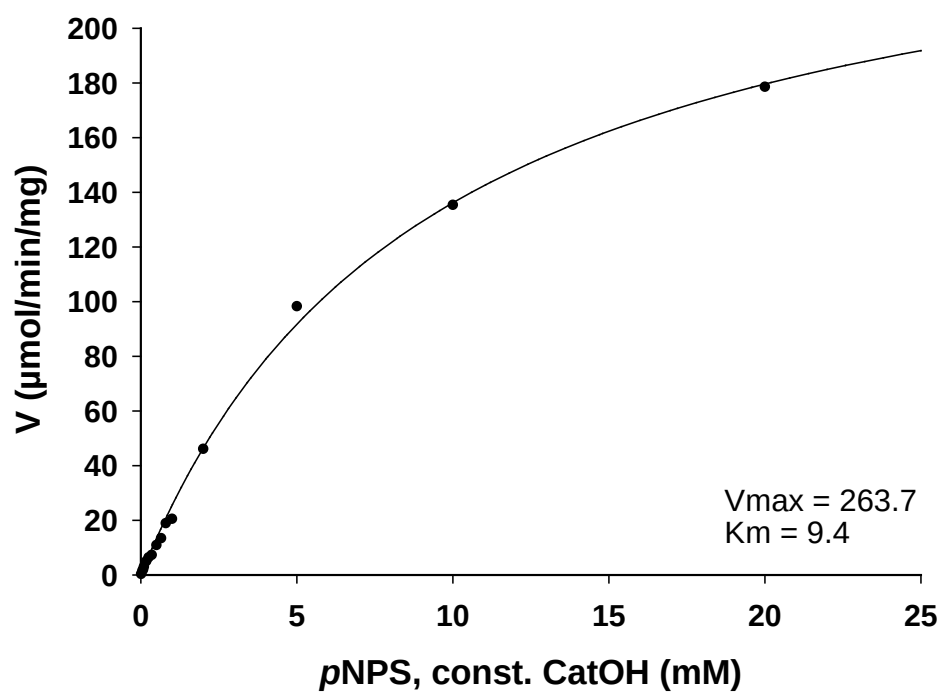

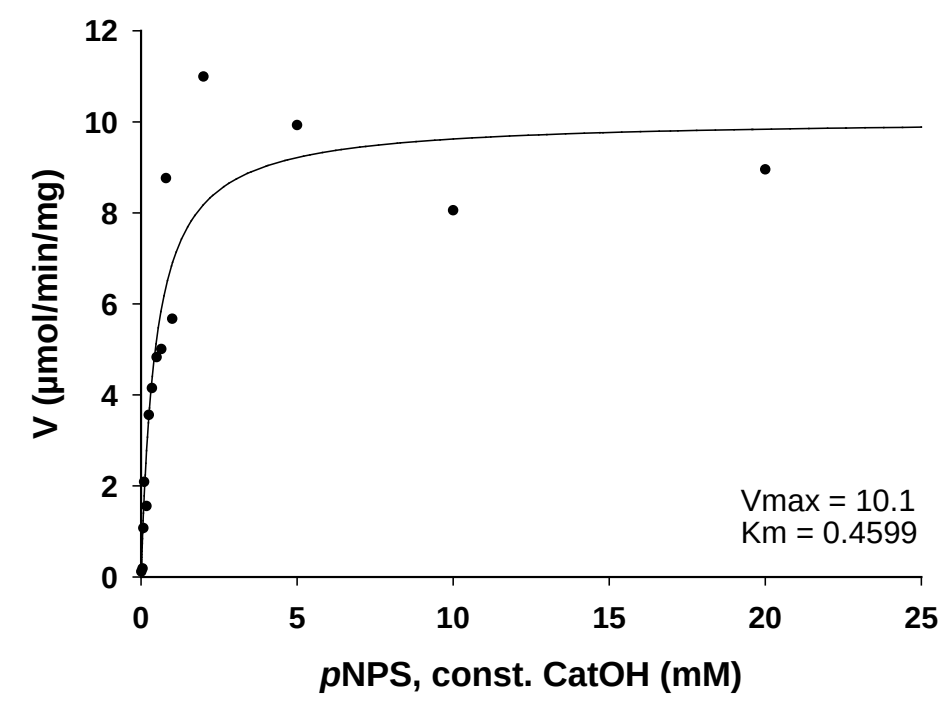

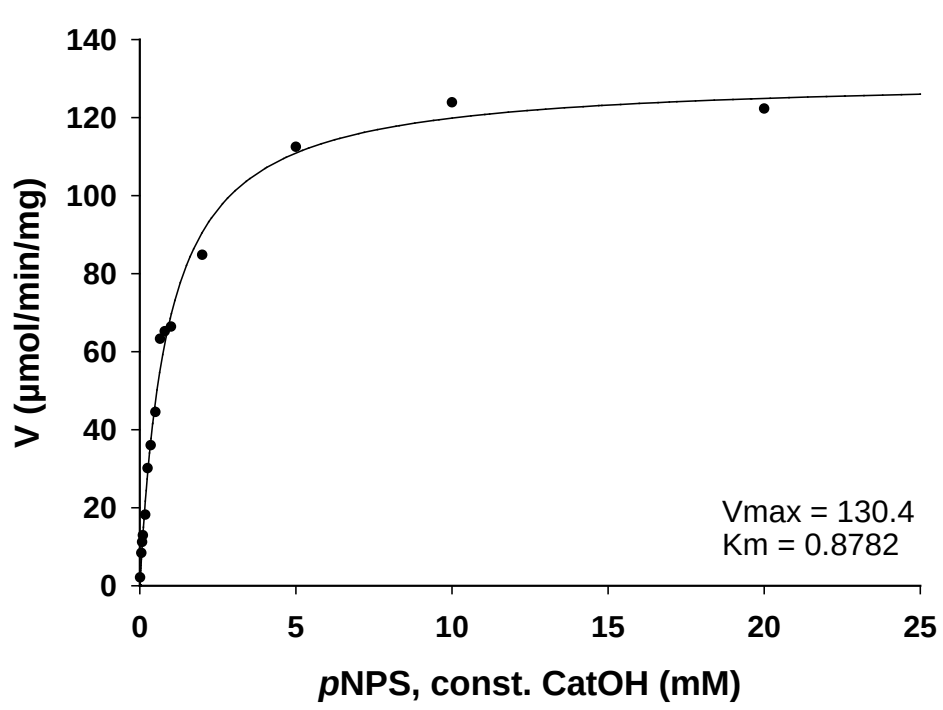

ASSTC

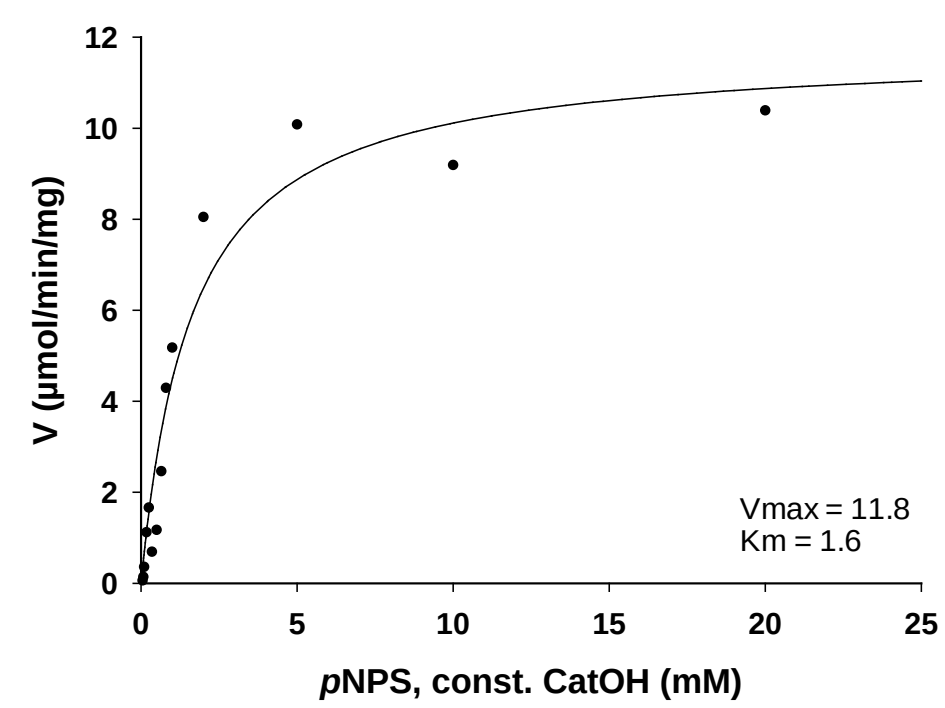

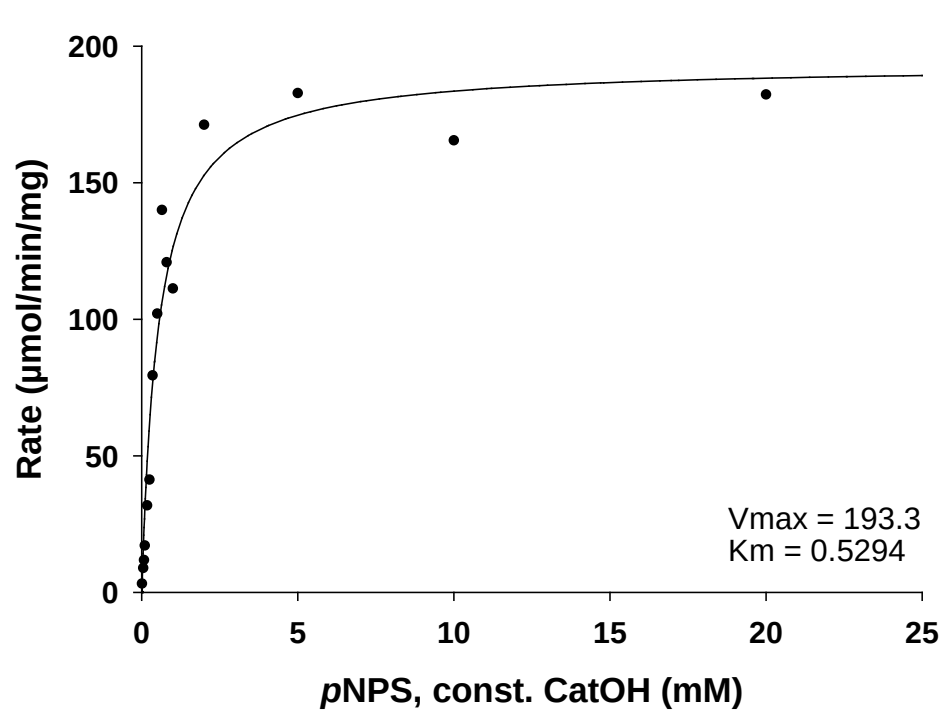

Fig. S7. Apparent kinetic parameters of ASSTA, ASSTB, ASST*Heff*, ASST*Dor*, ASST*Dfor*, ASSTC, and ASST*Ddeh* for *p*NPS. Reaction conditions: Tris/HCl buffer (50 mM, pH 8.0), *p*NPS (0-20 mM), catechol (**2**) (100 mM) and the purified ASST (50 nM)

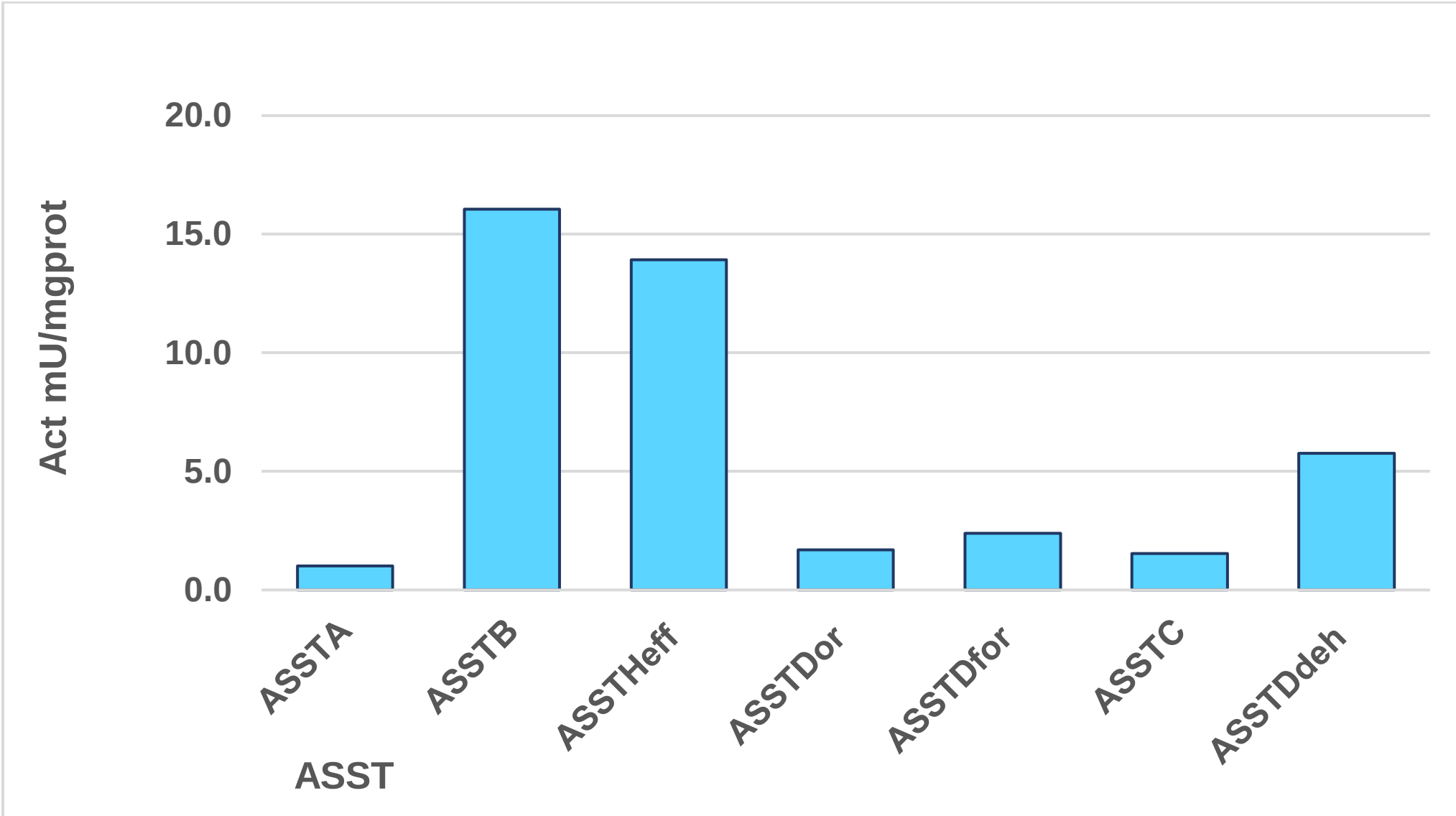

Fig. S8 Activity displayed by each enzyme in presence of 10% 2-propanol in the absence any other acceptor substrate. Reactions conditions: Tris/HCl buffer (50 mM, pH 8.0), *p*NPS (1 mM), 2-propanol (10%, v/v) and the purified ASST

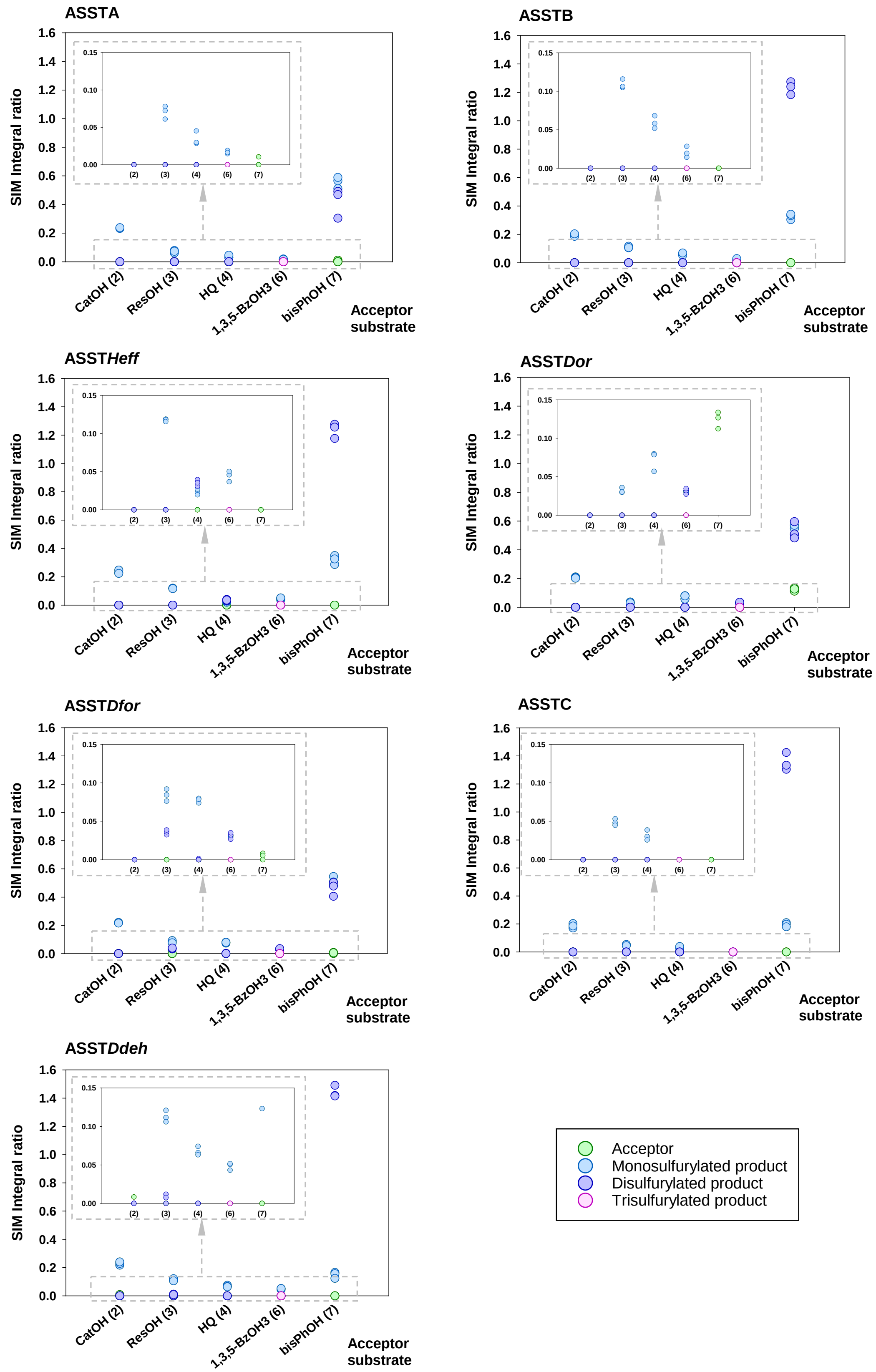

Fig. S9 Polysulfation screening of di- and trihydroxy aromatic compounds catalyzed by ASSTs. Coloured dots show the SIM integral ratios (target/buffer) of the substrate and the possible products in the biotransformations. Reactions were carried out in buffer PIPES 20 mM, pH 8.0; acceptor (2, 3, 4, 6, or 7) 0.5 mM; *p*NPS 1.5 mM; and purified ASST.

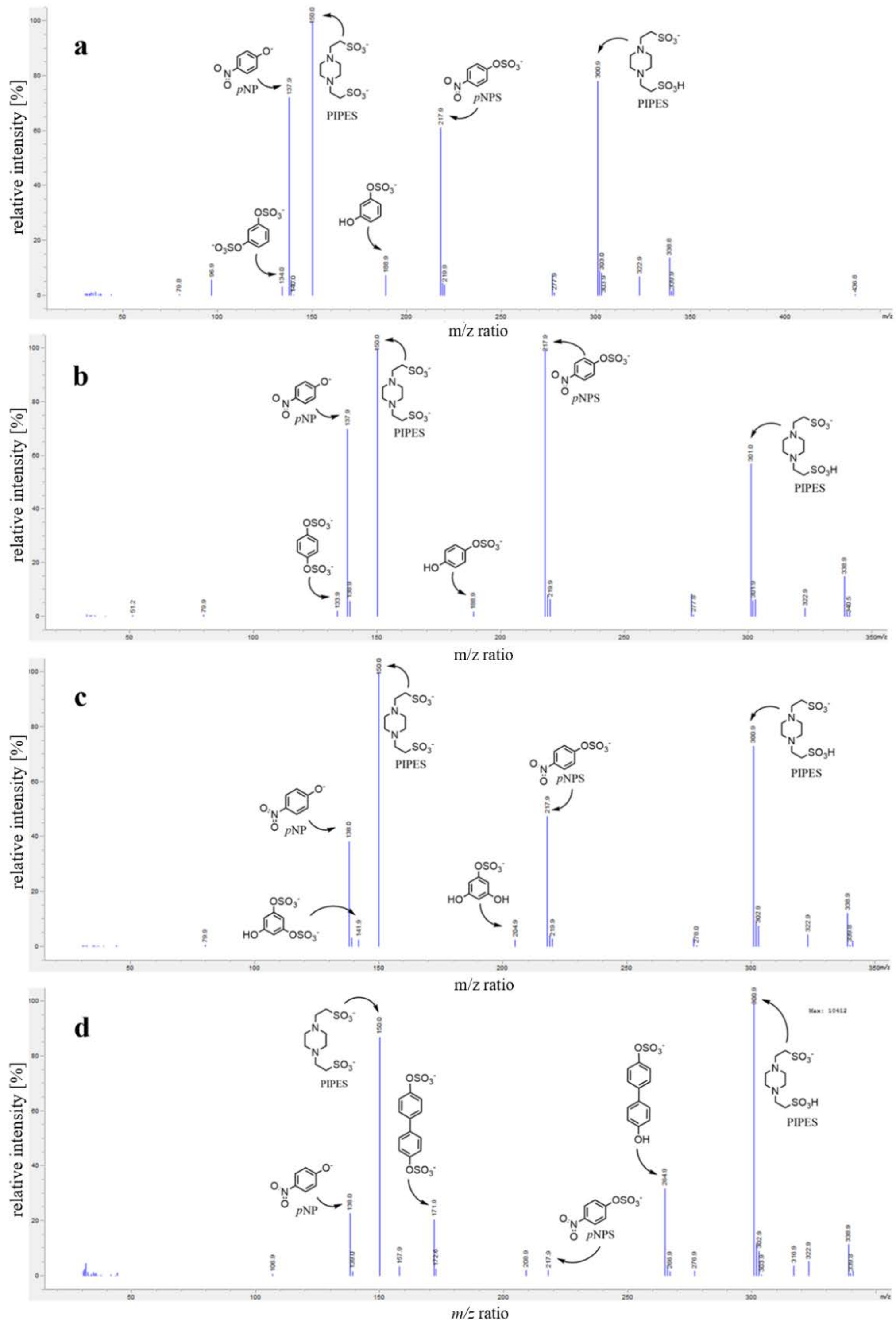

Fig. S10 Examples of mass spectra obtained in FIA-MS analysis of disulfation reactions with ASST. (**a**) Conversion of resorcinol (**3**) to the mono- and disulfate derivative (*m*/*z*: 188.9 and 134.0 respectively). (**b**) Conversion of hydroquinone (**4**) to the mono- and disulfate (*m*/*z*: 188.9 and 134.0 respectively). (**c**) Conversion of 1,3,5-benzenetriol (**6**) to the mono- and disulfate (*m*/*z*: 204.9 and 141.9 respectively). (**d**) Conversion of 4,4'-dihydroxybiphenyl (**7**) to the mono- and disulfate (*m*/*z*: 264.9 and 171.9 respectively).

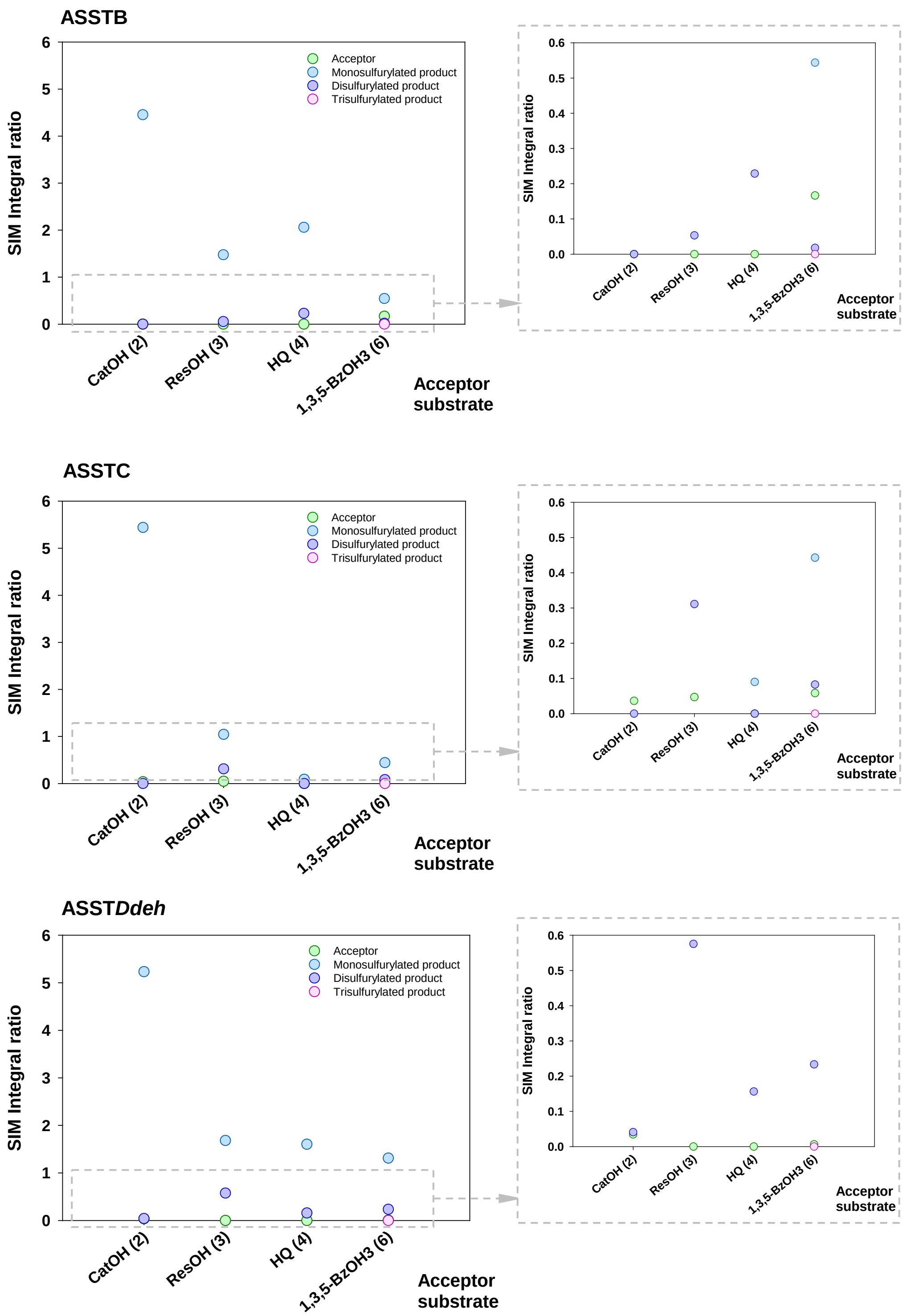

Fig. S11 Polysulfation screening of di- and trihydroxy aromatic compounds catalyzed by ASSTB, ASSTC and ASST*Ddeh* during a long period of time. Colored dots show the SIM integral ratios (target/buffer) of the substrate and the possible products in the biotransformations. Reactions were carried out in buffer PIPES 20 mM, pH 8.0; acceptor (**2**, **3**, **4**, or **6**) 10.0 mM; *p*NPS 30.0 mM; and purified ASST. Reactions were shaken at 650 rpm during 16 h.

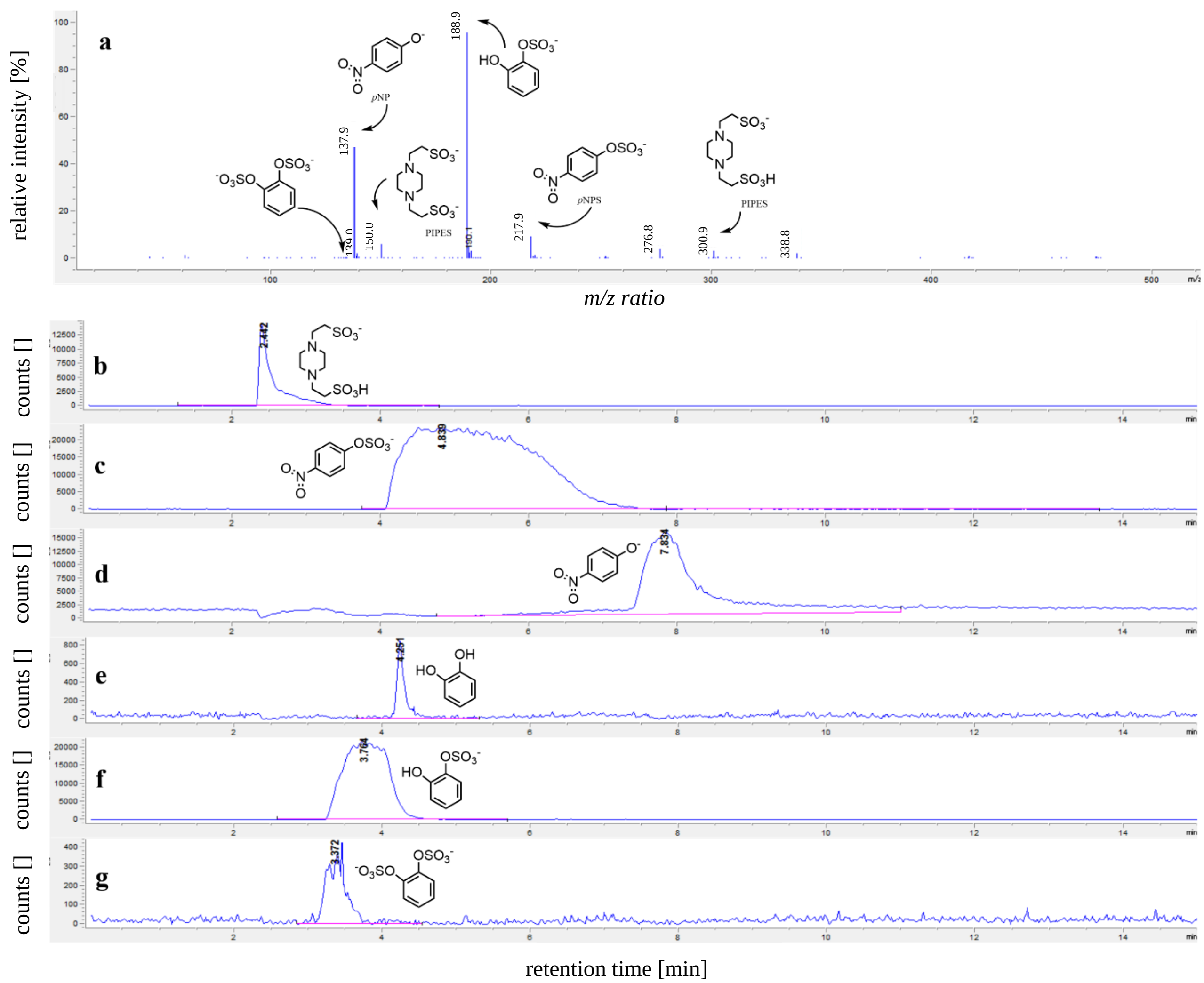

Fig. S12 (**a**) Mass spectrum obtained in 16 h disulfation reactions catalyzed by ASST*Ddeh* with catechol (2) as acceptor substrate, identifying important observed species. (**b-g**) Extracted ion chromatograms showing the elution peaks of different species, i.e., (**b**) PIPES (*m*/*z* 301), (**c**) *p*NPS (*m*/*z* 218), (**d**) *p*NP (*m*/*z* 138), (**e**) catechol (2, *m*/*z* 109), (**f**) catechol monosulfate (*m*/*z* 189) and (**g**) catechol disulfate (*m*/*z* 134).

Plasmid sequences

<u>ASSTA *D. hafniense* as cloned in pET26b *in silico*</u>

Name: pET26b-PelB- ASSTA-His

Restriction Enzymes

5’: *Sac*I

3’: *Sal*I

5’ Flanking: A

3’ Flanking: None

Gene sequence ordered

ATGAATCCGATCAAAAGTGAACAGATTCCCCATATTATCCATAGACAAAAGGATTTGGAAGAAGCTTTTCTGGCTG
AATTCTCAGCGGGTCACTATACGCTGGAGAATCCGCTGGTTAAGCTCAACCCTTATGATATTTGCCCCTTAACGGCC
ATGGTCTTATTTGAAACACCCGTAGCCACCGAGGCAACCATAATCGTTCGTGGCAAAGAGCACCCCGGAGATATCC
GGCATACCTTCCCCGCCGATAAAAAACATATTCTGCCGGTTTATGGTCTCTATGCCGATTACGAAAATAAGATCGA
AATCGTCCTGGCCAATGGGCAGAAGAACACCATAACCCTTAAGACCGAGCCCCTTCATCCCGATGTCCCGGTGGCC
ACCTCGATCAAGACCACCCCGGAATACATGGGCAACAACCTGATGTTTCTGACGGCAGCGATGAAGGCTATGCCTG
TAGGCTATGACTATGCCGGAGAAGTTCGCTGGTATGCCACAAGGAACTTTGCCTTTGATCTCAAGCGTATGCCCAA
TGGACATATTCTCATTGGTACGGAGCGTTTGGTCAAATTGCCCTATTTCACCACAGGTCTATATGAAATGGCCTTTA
GTGGGAAGATATTCAAAGAATACCGTCTATCCGGCGGATACCACCATGATCAATTTGTCATGGAAGATGGCAACAT
TCTGGTGCTTACTTTTGATTTCTACTCAGGTACGGTTGAAGATATGTGCGTGCTCCTGGATGCCATAACGGGAGAAA
TTCTCAAGTCATGGGACTATAAAAGGGTCCTGCCCCAGGATGTAGCCGGTTCCGGAAGCCAGGATGCCCACGATTG
GTTTCATAATAACGCCGTTTGGTACGATAAGAAGACACACAGCTTAAGCTTCTCCGGTCGTCACCAGGATGTGGTG
ATCAACCTTGATTATGATACAGGTGAGTTAAACTGGATCATTGGGGATCCTGAAGGATGGCCCCAGGACATGGTGG
ACAAATATTTCTTTACCCCGGTTGGGGAAGGGGAATTTGACTGGCAGTATGAGCAGCATGCTTGCGTCGTTTTACCT
GATGGGGATATCATGCTCTTTGATAACGGCCACTTCAGAGCGAAGAAAAAAGAGAATTACTTGCCCAACGGCCGG
AATTTCAGCCGTGGTGTGAGGTACCGTATTGATACCGAAAAGATGACCATAGAACAAGTATGGCAATATGGAAAA
GAGCGGGGCGCGGAGTTCTTCTCTCCCTACATTTGCAATGTGGAGTATTACAATGAAGGCCATTACCTGGTTCACTC
CGGCGGCATCGGCTATGAAAACGGTGAAACCTGCGAAGGTATGGCAGTTATGAAAGTCCTGCAACCGGAGTTTAA
GGATAGTGTGTTTACCTTCAATTCCATTACCTGTGAGCTTAAAGATGACGTGCTGATGTATGAGTTGCAAGTACCGG
CCAATTGTTACCGGGCTGAAAAATTGCCCCTCTACTATGCCCACGAAACGGCTGAATTAGGTGCGGGCGAAATACT
GGGCAATTTAATTGAGACCCAGGAGACAAAGATGAAGATCAAGGCTGTGGAGACAGGTGAAAGAGTGCCGGATCA
TTATGAGGCATCCATCACAGAAGAAGAGGATCGGGTTCTCTTTAACGCCATCTTCGAGGCCGGGGAAATGGCTCAG
CTGCTTTTGGTGGACGGAGACGGCGGGGTAAAGAGATATCCTGTCAATACTGTGCCTCAGGCCTTCCAAGCCATGT
GTGTAGGGACGTTCCAGAAAGCTGACCCCCGCAATATCGATGTTTATATCAACAAGACCGGATTATCCGGAAAATA
TCAAGTAAAGCTCATCGCAGAAGAAAAACTCTATGAGACCGGAGTGTCTATTACAGCT

ASSTB *D. hafniense* as cloned in pET28a *in silico*

Name: pET28a-ASSTB-His

Restriction Enzymes

5’: *Nco*I

3’: *Sac*I

5’ Flanking: CA

3’ Flanking: AA

Gene sequence ordered (optimized for *E. coli*)

CTGCGTACCTATCTGAATACCGAAAAACATCTGATTACCCTGCAGGCAGAAAGTGAAGAACGTTTTCTGGCCGAAC
TGCGTGCCGGCAATTATACCGCAGAAAGTCCGCTGGTTGTTAAAAATCCGTATATTATTAACCCGCTGGCCGCCGTT
ATTTGTTTTAATACCGATGAAGAAACCACCGCCGAAATTACCGTTAAAGGCAAAGCCATTGAAGGCGATCTGAGTC
ATACCTTTGCCGCCGCCAAAGAACATGTTCTGCCGGTTTATGGCCTGTATGATGATTATGTTAATACCGTTGTGATC
AAACTGAGCAATGGCAAAACCAGCGAAGTGAAAATTGAAGTGGAAGAACTGAATGTTAACAAAGCCCTGTATTGC
CGTACCACCCCGGAATATTTTGGTAAAGATTTTATGCTGATCAGCACCACCACCCCGCTGATTGAAAGCGCCCGCA
CCGCCGGCTTTGATTATGCAGGCGATCTGCGCTGGTGTATTACCAATCTGCAGAGTTGGGATATTAAGAAACTGGA
AAATGGCCGTCTGCTGTATACCAGCCATCGTACCGTGCAGAAACCGTATTATAATGTTGGCGTTATGGAAATGGAT
TTTTGTGGCAAAATCTATAAGGAATACCGTCTGCCGGGCGGCTATCATCATGATGCCGTGGAACTGGAAAATGGTA
ATATTCTGGCAGCCAGTGATAATGATTTTAATGATAGTGTTGAGGACTTCGTTGTTGAAATTGAACGTGCCACCGGT
GCAGTTATTAAGAGCTGGGATCTGCAGAAAATTCTGCCGCGCGGTCAGGGCAAAGCAGGCGATTGGAATCATCAT
GATTGGTTTCATAATAACGCAGTGTGGTATGATAAACCGACCAATAGCATTACCATGAGCGGTCGCCATATGGATG
CCGTGATTAATTTTGATTATGATAGCGGCGCCCTGAATTGGATTCTGGGCGATCCGGAAGGTTGGAGCGAAGAATG
GCAGAAATATTTCTTTAAAAACGTGACCAAGGGTGACTTTGATTGGCAGTATGAACAGCATGCCGCCCGTATTCTG
CCGAATGGTGACGTTTTTCTGTTTGATAATGGCACCTATCGTAGTAAAAATGAAGCAACCCGCGTTGATCCGGAAC
AGAATTTTAGTCGTGGTGTTATCTATCGTATTGATACCGATAAAATGGAAATCGAACAGGTGTGGCAGTATGGCAA
AGAACGCGGCGCCGAATTTTATAGTCCGTATATTTGTAATGTGGACTATTATGGTGAGGGTCATTATATGGTGCATA
GCGGCGGTATTGCAACCTATCGCGGCAAACATACCGATGGCCTGGGTGCAATGCTGCTGAATAAGTATAAAGATGA
ACATATCCACCTGACCCTGGAAAGCATTACCGTTGAAGTTCAGAATGATCAGCTGAAATATGAACTGAAAGTGCAG
GGCGGTAATTATTATCGCGCACGCCGCGTTAGTCCGTATGATGAAAAAACCAATCTGGTGCTGGGTAAAGGTGAAC
TGCTGGGTGGTTTTGGTGTTACCCCGGAATTCATGAAAGTTAATTTTAAAGACGCCGAAACCGAACTGAGTGAAAA
ACATAATCTGAATGTTATCCTGGAAGAAGATCGCCTGGCAATTCGCGCCAGCTTTCGTGAAGGCAGCCAGGTTTTT
CTGGAACTGAAAGGCGCCGAACAGAGCAAATTTTATAATATTCCGACCGAAGTTCACGATGTGACCGCCGCCTGCG
TTAGTTTTGAAGAACAGAATGATAATGACTTCCAGTTTTATGTTAGCCGCGAAGGCCTGAGTGGTGAATTTGGTATC
TATCTGAATATTGATAGCAAGCGCTATGATACCCATCTGAGTGTGAAACTG

ASST *D. dehalogenans* as cloned in pET28a *in silico*

Name: pET28a-ASST*Ddeh*-His

Restriction Enzymes

5': *Nco*I

3': *Sal*I

5' Flanking: GA

3' Flanking: -

Gene sequence ordered (to be optimized for *E. coli*)

ATGATGAATCCGATTAAAAGCGAACAGATTCCGCATATTATTCATCGTCAGAAAGATTTTGAAGAAGCATTTCTGG
CAGAATTTAGCGCAGGTCATTATACCCTGGCAAATCCGCTGGTTGAACTGAATCCGTATGAAATTTGTCCGCTGACC
GCAATGATTCTGTTTGAAACCCCGGTTAGCACCGAAGCAACCATTATTGTTCGTGGTAAAGAACATCCGGGTGATA
TTCGTCATACCTTTCCGGCAAATAAAAAACATATTCTGCCGATTTATGGTCTGTATGCAGATTATGAAAATAAAGTT
GAAATTGTTCTGGCAAATGGTCAGCGTAATACCGTTACCATTAAAACCGGTCCGCTGCATCCGGATGTTCCGGTTGC
AACCAGCATTAAAACCACCAGCGAATATATGGGTAATAATCTGATGTTTCTGACCGCAGCAATGAAAGCAATGCCG
GTTGGTTATGATTATGCAGGTGAAGTTCGTTGGTATGCAACCCGTAATTTTGCATTTGATCTGAAACGTATTCCGAA
TGGTCATATTCTGATTGGTACCGAACGTCTGGTTAAAATGCCGTATTTTACCACCGGTCTGTATGAAATGGCATTTA
GCGGTAAAATTTTTAAAGAATATCGTCTGCCGAGCGGTTATCATCATGATCAGTTTGTTATGGAAGATGGTAATATT
CTGGTTCTGACCTTTGATTTTTATAGCGGTACCGTTGAAGATATGTGTGTTCTGCTGGATGCAGAAACCGGTGAAAT
TCTGAAAAGCTGGGATTATAAAAAAGTTCTGCCGCAGGATGTTGCAGGTAGCGGTAGCCAGGATGCACATGATTGG
TTTCATAATAATGCAGTTTGGTATGATCGTAAAACCAATAGCCTGAGCCTGAGCGGTCGTCATCAGGATGCAGTTA
TTAATATTGATTATGAAACCGGTGAACTGAATTGGATTATTGGTGATCCGGAAGGTTGGCCGCAGGATATGGTTGA
TCAGTATTTTTTTACCCCGGTTGGTGAAGGTGAATTTGATTGGCAGTATGAACAGCATGCATGTGTTGTTCTGCCGG
ATGGTGATATTATGCTGTTTGATAATGGTCATTTTCGTGCAAAAAATAAAGAAAATTATCTGCCGAATAGCCAGAA
TTTTAGCCGTGGTGTTCGTTATCGTATTGATACCGAAAAAATGACCATTGAACAGGTTTGGCAGTATGGTAAAGAA
CGTGGTGCAGAATTTTTTAGCCCGTATATTTGTAATGTTGAATATTATGATGAAGGTCATTATCTGGTTCATAGCGG
TGGTATTGGTTATGAAAATGGTGAAACCTGTGAAGGTATGGCAGTTATGAAAGTTCTGCAGCCGGAATTTAAAGAT
AATGTTTATACCTTTAATAGCATTACCTGTGAACTGAAAGATGATGTTCTGATGTATGAACTGCAGGTTCCGGCAAA
TTGTTATCGTGCAGAAAAACTGCCGCTGTATTATGCATATGAAACCGCAGAACTGGGTGAAGGTAAAATTCTGGGT
AATCTGGTTGAAACCCAGGAAACCAAAATGAAAATTAAAGCAGTTGAAACCGGTGAACGTGTTCCGGATCATTAT
GAAGCAAGCATTACCGAAGAAGAAGATCGTATTCTGCTGAATGCAATTTTTGATGCAGGTGAACTGGCACAGCTGC
TGCTGGTTGATGGTGATGGTGGTGTTAAACGTTATCCGATTAGCACCGTTCCGCAGGCATTTCAGGCAATGTGTGTT
GGTACCTTTCAGAAAGCAGATCCGCGTAATATTGATGTTTATATTAATAAAACCGGTCTGAGCGGTAAATATCAGG
TTAAACTGATTGCAGAAGAAAAACTGTATGAAACCGGTGTTAGCATTACCGCA

ASST *D. formicoaceticum* as cloned in pET28a *in silico*

Name: pET28a-ASST*Dfor*-His

Restriction Enzymes

5’: *Nco*I

3’: *Sal*I

5’ Flanking: GA

3’ Flanking: -

Gene sequence ordered (to be optimized for *E. coli*)

ATGAACGCAATTAAATCTGAACAGGTTCCTCATATTATTCATCAACAGGAAAAGCTGGAAAAAGCATTCCTCGCTG
AATTTGCTGCAGGTGGTTACACTTTATATAATCCTCTGGTCAAAATCAATCCGTATGGGATTTGTCCTTTAACTGCG
ATGGTGTTATTCGAAACACCGGTCGCCACAGAGGCCACAGTGGTAGTGCGGGGCAAAGAACATCTGGGTGACATA
CGTCACACATTCCCAGCTGGAAAAAAACACATACTGCCAATCTATGGTCTTTACGCCGACTATGAAAATAAGATCG
AATTGATAATGGCTAATGGCCAGAAAAATACCATTAAAATCCAAACTGAACCGCTTCATACAGATGTACCCGTGGC
TACTTCTATAAAGACCACACCAGAATATATGGGTAACAATTTGATGTTTTTGACCGCCGCGATGCGCTCCATGCCTG
TGGGTTATGATTACGCAGGGGATATTAGATGGTATGCAACCAAAAATTTCGCCTTTGACTTGAAACGCCTGCCCAA
TGGCCACATTCTGGTGGGAACCGAACGTTTAGTCAAAATGCCCTATTTTACAACTGGTCTTTACGAAATGGCGTTCT
CTGGGAAAATTTTTAAAGAATATGTTCTGCCAAGCGGCTATCATCACGACCAGTTCCCGATGGAAGACGGTAATAT
ACTGGTGCTCACCTTTGACTTTTACAGCGGGACAGTTGAGGATATGTGTGTGTTGCTTGACGCGAAAACTGGAGAA
GTGTTAAAGTCTTGGGACTATAAAAAAGTCCTGCCACAAGATGTGGCGGGTAGTGGTTCTCAGGATGAACATGATT
GGTTTCACAACAACGCAGTATGGTATGATAAGAAAACTAACTCACTGTCTCTTTCAGGGAGACACCAGGATGCAGT
GATAAATATCGACTATGAGACTGGCGAATTAAATTGGATAATTGGTGATCCGGAGGGTTGGCCTAAAGAAATGGTT
GATAAATACTTCTTTACCCCGGTTGGAGATGGTGACTTCGATTGGCAATATGAGCAGCACGCTTGCGTGGTACTGCC
GGATGGTGACATTATGCTGTTTGATAATGGGCATTTCCGTGCAAAAAACAAGGAGAATTACTGGCCAAATTCCAAA
AACTTTAGCCGCGGAGTACGATATCGTATTGATACCGAGAAAATGACCATAAAACAGGTCTGGCAATACGGCAAG
GAACGTGGCGCAGAGTTCTTCTCGCCATATATTTGCAACGTGGAGTATTACAACGAAGGGCATTATCTGGTTCATTC
CGGAGGAATTGGTTATGAAAACGGGAAGCCTTGCGAGGGCATGGCGGTTATGAAAACGATGCAGCCTGAATTTAA
GGATAACGTGTATACTTTTAACAGCATTACATGTGAACTGAAAGATGACGTTCTGATGTATGAATTGCAGGTGCCT
GCCAATTGCTATCGTGCCGAAAAACTGCCACTTTACTACGCCAACGAAACGGCCGAACTGGGCGCCGGTGAAATTC
TAGGAAACTTGATTAAGACTGAAGAGACTAAAATGAAAATCAAAGCTGAAGAAACTGGCGCCCTCGTCCCAGATC
ATTTCCAGGCCAGCATTACGGAAGAAGAGGATCGATTTCTTTTTAATGCCATTTTCGACGCAGGAGAGATGGCCCA
GTTGCTGTTAGTTAACGAAGCTGGAGAAATTAAAAGATATCCAATTAACACTGTGCCGCAAGCGTTCCAGGCAATG
TGCGTCGGCACTTTTCAGAAAGCAGACCCTCGTAATATTGATACGTTCATAAATAAAATTGGTCTGTCTGGTAACTA
TCAGGTCAAACTGGTTGCTGAAGAAAAGGTATACGAGACAGGGGTTACAATCAAGGCA

<u>ASSTC *D. hafniense* as cloned in pET28a *in silico*</u>

Name: pET28a-ASST*Dhaf*03-His

Restriction Enzymes

5': *Nco*I

3': *Sal*I

5' Flanking: GA

3' Flanking: -

Gene sequence (<u>to be optimize for *E. coli*</u>)

ATGGTGGATTATGAATATTGTCCCCATATTATCACGAAACAGAATGAGTTAGAAAAAGCATTTATGGAGAAATTCG
AGGCTGGGAATTATTCGCTGGAAAATCCATTAGTGATCCTGAATCCGTATGGCGTGGCACCGCTAACAGCAATGAT
CCTCTTTAATACCCCTATCGCTTGCGAAGCGAAAATAATTGTGAAAGGAAAGGAACATCCCGGCGATATTCAACAT
ACTTTCCCAGCAGCGACGCGACATATACTGCCGGTCTATGGACTGTATGGAGGTTATAAAAATACTATTAAGATTA
TCCTTTCCACGAGTTCGTCAAATACTATCACGATTCAGACCGAACCATTGCCGAAGAGTGTTGCCATTCCCTCGAGC
ATTAAGACTACCTCGGAATATATGGGAAACAACCTGATGTTCCTCTCTCCGTCAATGGCTAGTCTTACTGTGGGCTA
TGATTATGCCGGGGATGTACGTTGGTATTGCTCATTGAACGTCTGCTTCGATCTCAAACGTATGCCGAACGGCCACT
TGCTGATTGGCACCGGACGCCTTGTGAAAGTACCGTACTATACAAGTGGCTTATATGAAATGGCGTTTAGCGGCAA
AATATTCAAAGAATTTATCCTACCGGGTGGCTACCATCATGATCAGTTCGTGATGGAAGATGGCAACCTGCTGGTT
CTTACTCAAGACTTCTATGCCGACACCGTAGAAGATGTATGTGTACTGATCGATCCTCACTCTGGTGACATTCTGAA
GAAATGGGATTTTAAAAATATCCTTCCCCAAGATGTGGCAGGCTCAGGAACACAAACAGCGCACGATTGGTTTCAC
AACAATGCAGTATGGTATGATAACAGAACTAACTCTCTGACTTTGTCTGGACGACACCAGGATGTGATCATTAACA
TTGATTACGAAACTGGGGGACTCAACTGGATGATTGGGGATCCTGAAGGATGGCCGCAGGAACTAGTGGATAAAT
ACTTTTTTACCCCTGCCGGCGACGGCGAATTCGATTGGCAGTATGAACAGCACGGTTGCGTTGTGCTGCCTGACGG
AGACATCATGGTATTCGATAATGGTCATTATAGGTCTAAAAACAAAGAGAATTATCGCTTGAATAAAGACAATTTT
TCTCGCGGTGTGCGCTACCGCATAGATACGGAGCGTATGACCATTCAGCAGATTTGGCAGTATGGGAAGGAGCGCG
GCGCGGATTTTTTCTCTCCATTCATTTGCAATGTAGAATATTATGATGAGGGACATTATATGGTACATTCTGGAGGT
ATAGGCTACGAAAATGGGAAACCCTGTGAAGGCTTGGCAGTGGGGAAAAGCAAAAATCCGAAATTCAAAGACAAT
GTTTATACACTCAATTCGATCACGTGCGAACTGGTAAATGATGAATTGGTGTATGAACTGCAAGTTCCGGCAAATTT
TTATCGCGCCGAGAAATTACCAATTTATTACGCAAACGAGGTCGCGGAGCTGTGTGAAGGGCAGCGGCTCGGACA
GTTCGTGGAAACACAAACCACACGTATGAAGATCAAGGCACTCGAGACGAAAGAGCCGATCCCAGAACATTATCA
AGCTAAGATTGTGGAAGAAGAGGATAGATTCTGCCTGAACGCAATTTACGAGAGTGGGGAGACAGCTCAACTGCT
GCTGGTCTCAGAATCAGATGAAATTTTGAGATATCCAATTAATACAGTGGCTCAGGCGTATGGTGCCATGTGTGTG
GGCACCTTCCAAAAATCTGATCCTCGGAATGTAGACACTTTCGTGACCAAGACAGGGTTGTCCGGAAAATACCAAG
TCAAACTGGTCGCGGAGGACAAGATATATGAAACCGGTGTGACAATTTCGGCT

ASST *D. orientis* as cloned in pET28a *in silico*

Name: pET28a-ASST*Dori*-His

Restriction Enzymes

5': *Nco*I

3': *Sal*I

5' Flanking: GA

3' Flanking: -

Gene sequence ordered (to be optimize for *E. coli*)

ATGTCGGTAAAGTTTAAAAGTTTTGATCACATTATCACCGAACAGTACCAGGCAGAGCAGGCATTCTTAACAGAAT
ATCAAAAAGGTGAATACAGCTTTAATAACCCGTATGTGAAACTTAACCCTTATATTATTGCCCCATTAACCGCACTT
GTCATGTTCAAAACTGCGCAGCCTACCACTGTTACCATCACCGTCAAAGGAAAAGACAAGAATGGAGATATATGCT
TTAATTTTCCAACGGCCATAGAACACCTGATCCCAGTTTATGGGTTGTATGCCAACTATGCCAATAAAGTAGAACTT
GCGTTGGGTGATGGGAAAACAAACGTTATCACCATACAGACGGAAGCTGCCCCTGAAATTGTGAAACTTCCAACCC
AGATTAACACGACAGCGGATTATTTTGAGGACAATATTATGTTCGTTAGTCCAACAAGTACTGCCGCTACCGCGGG
ATATGACTATAATGGGGATGTGCGCTGGTATGGTTCCCTGAATTTTGCGTTTGATATCAAGAGAGCAAAGAATGGA
CGTCTTCTTTTAGGAACACACCGCCTTGTCACGCCTCCTTACCACACAACCGGCCTTTATGAAATGGGCATGATCGG
TAAAATTTATAAGGAGTATCGGTTACCAAGTGGTTACCACCATGACCAGTTTGAGATGGAAGATGGTAACTTATTA
ATCCTGTCCCAAGATTTACCGCGGGGTACAGTTGAAGATATGTGTGTATTAGTAGATAGAAAGACGGGTCAGATCA
TAAAGAGTTGGGATTACCAAAAAGTGCTTCCGACGAACGCCGGGGGTTCTGGGTCGCAGGACTCCCACGACTGGTT
TCACAATAATGCCGTTTGGTACGATAAAAAGACCAACAGTCTCACTTTGTCTGGCAGACACCAGGACATCATAATT
AATCTGGATTTTGAGACCGGTGCACTGAACTGGATAATAGGGGATCCCGAAGGGTGGCCAAAAGAATTGGTCGAT
AACTATTTTTTTAAGCCGGTAGGTAAAGGTGAATTCGACTGGCAGTACGAACAACATGCATGTGTGGTTTGCCCGA
ATGGTGACATAATGGCCTTTGATAATGGGCATTGGCGCTCTAAAATTAAAGAGAATTACGTCACTGCTAACGATAA
TTTTTCGCGAGGGGTTCGTTATCGTATCGATACCAAGAAGATGGAGATCGAACAGGTGTGGCAATATGGAAAGAA
AGAGGCGCGGAATTTTTCAGCACGTACATTTGTAATGTGGAGTACTACGATGAGGGGCACTACTTAATCCATTCTG
GCGGCATCGGTTCCATTGATGGCGAAGCCCTTAATAAACCCCCGGTGCAGTTTAGGGGAGAAGATGAAAAAAAGG
TGGTATTTAACTCAATTACCGTAGAACTGAAAGATGACGTGATGATGTATGAAATGCAGCTGCCTGCTAACTATTA
TCGTGCCGAAAAACTGAAGTTATACGCCGCGGAAGATGTTCTGACGCTGGGTAAAGGGGAACTTCTGGGGACATTA
GGAGTGACCGAAGAGTTCACCACGATACCCCCGGCAGAAGAAGGCGGGATGGTTCCGGAAAAATACAATGTAAAA
CTGGGCTTAGAAGAAGATCGGCTTATTTTTAAGGCCACATTTGAAAAAGGTCAGATGGTGCTATTACAGCTCGAAG
GTGAAACAACGCATTCATATTTTGTGCCTACTACCAAGCGACCATTTTTGGCCATGTGCGTCGGCACGTTTCAGGAA
TCAGATGATCGTGCTGTAGAATTCCCCGTTTCTCGAGAAGGACTGGCTGGAGGTTTTCGGGTTTCTCTAATTGTGGA
CGCATATAAGTATGAAACCGGGGTCACTATAAAACTA

ASST *H. effluvii* as cloned in pET28a *in silico*

Name: pET28a-ASSTHeff-His

Restriction Enzymes

5’: *Nco*I

3’: *Sal*I

5’ Flanking: GA

3’ Flanking: -

Gene sequence ordered (to be optimize for *E. coli*)

ATGATCCGTTATGAGAAAAAGAATTCACTCATTACACAGCAGGCGGAAGCAGAACAGAAGTTTTTGGAAACATTTG
AAGCTGGGGCCTACACCGCGAGTAGTCCGCTGGTTGTGCGTAATCCTTATCTGATTAGCCCGCTTTCCGCAATGATC
CTGTTTAAAACAGCCGTTAAACAAGAGGTAACACTGACGGTGAAAGGGAAAGAGCCGGAAGGTGATATCTCTCAT
ACTTTCCCGGCAGACACAATTCATATTTTACCAGTGTATGGTCTATACGCAGACTGTGAAAACACAGTTGAACTTAT
CTTATCTGGGGGGGAACGAGAGACAGTCATGATCAAAACGGAACCGCTGCCTCCTGAGGTCCCTGTTCCTACTTTT
TGTAAAGGTTCTGGCGCGCATATGGGGGACAATGTTATTTTCCTCACGCAGACATCCAAAGCCGACGCGTTGGCCT
GCGATTACCGGGGCGATGTTAGATGGTATCTGACAGTTAATGTGTGCTTCGATATGAAACGCCTGGCAAACGGTCA
CCTTTTGGTGGGAACCGATCGCTTGATTAAGCTGCCGTACTACGTAAGCGGTGTGTATGAAATGGGTGTTCATGGA
AAGATTTACCGCGAATATCGTCTGCCTGGAGGTTACCACCATGATACGTTTGAAATGGAAGATGGTAATATTCTGA
TGTTGAGCCAAATACCGGACCGGGATACGGTGGAAGACGTCCTGGTCCTCGTAAATCGCCAGACTGGCGCGATTGT
TCGAACATGGGATTACCGCGAAATTCTGCCCTACGACTGTCAGACCACATGGTCAGGGTCAGCGAGCGCTCACGAT
TGGTTTCATAATAATGCCGTTTGGTATGATAAGAAAACGGATTCGATTACACTCTCTGGCCGTCATCAGGATGCTGT
CATCAATATCGATTTTAAAACAGGTGCCCTCAATTGGATTCTTGGAGACCCAGAAGGGTGGCCCGAAGAATATGTA
GAAAAATATTTTTTCCGTCCGATATCAGAGCCGTTCGAGTGGTCCTATGAACAGCATGGAGTGGTCGTATGTCCGG
ATGGAGACATCATGATGTTCGATAATGGACACTACCGTTCTAAGCTGAAGGCACATTATTCAAAAGCTAAAGATTC
ATATAGCCGTGGTGTTCGGTATCATATCGATCGCGAAGCAAGGACTATTGAGCAGGTGTGGCAATATGGGAAAGA
GAGAGGCGCGGATTTCTTTAGCCCGTACATTTGCAATGTTGAATACTATGATGAGGGTCGGTACATGGTTCATAGT
GGCGGAATTGCGTACAAAGATGGTGAACCATTGGAAGGCTTAGGAAGCATGGATGGAACCGGTGAGGGGTGCACG
CTGAACGCCATAACATGCGAGCTGGTAAATGATGAGGTAGTGTACGAGTTGCACGTTCCCTCCAACGTTTTCAGGG
CCGAAAAATTGCCCATGTATTACGCAGGTGAAACCGCGGAATTAGGAGCAGGTAAAACCCTAGGCTCCATGTCAC
GTACGGGAGAATTCGAGACCGAAATTCCCGCAGTTTGCACAGGAGATCTGATACCGGAGCACTATGAAGCCTCTGT
AACGGAGGAAGAGGATCGTATATTGTTTAACGCGACCTTCGAAAAAGGTGAGCTCGCAATGCTTTTGCTGGAAGAA
GAGGACGGCAAAGCGCATCGTTATTATATAAACACTTCTGCCGCGAAAAATTTTGAGGCGATGTGCGTTGGCACTT
TCCTGAAGAATGATCCACGCAACGTAGATGTTTATGTCAATAAATGTGGGATGAGTAAGAACGTCAAAGTGAGGGT
TCTGTTAGAAGATAAAATCTACGAAACGGGTGTAGAGATCCGTATGGAA
